# The Flooring for Injury Prevention (FLIP) Study of compliant flooring for the prevention of fall-related injuries in long-term care: A randomized trial

**DOI:** 10.1371/journal.pmed.1002843

**Published:** 2019-06-24

**Authors:** Dawn C. Mackey, Chantelle C. Lachance, Peiwei T. Wang, Fabio Feldman, Andrew C. Laing, Pet M. Leung, X. Joan Hu, Stephen N. Robinovitch

**Affiliations:** 1 Department of Biomedical Physiology and Kinesiology, Simon Fraser University, Burnaby, British Columbia, Canada; 2 Department of Statistics and Actuarial Science, Simon Fraser University, Burnaby, British Columbia, Canada; 3 Department of Statistics and Actuarial Science, University of Waterloo, Waterloo, Ontario, Canada; 4 Clinical Quality & Patient Safety, Fraser Health Authority, Surrey, British Columbia, Canada; 5 Department of Kinesiology, University of Waterloo, Waterloo, Ontario, Canada; 6 New Vista Society Care Home, Burnaby, British Columbia, Canada; University of Cambridge, UNITED KINGDOM

## Abstract

**Background:**

Fall-related injuries exert an enormous health burden on older adults in long-term care (LTC). Softer landing surfaces, such as those provided by low-stiffness “compliant” flooring, may prevent fall-related injuries by decreasing the forces applied to the body during fall impact. Our primary objective was to assess the clinical effectiveness of compliant flooring at preventing serious fall-related injuries among LTC residents.

**Methods and findings:**

The Flooring for Injury Prevention (FLIP) Study was a 4-year, randomized superiority trial in 150 single-occupancy resident rooms at a single Canadian LTC site. In April 2013, resident rooms were block randomized (1:1) to installation of intervention compliant flooring (2.54 cm SmartCells) or rigid control flooring (2.54 cm plywood) covered with identical hospital-grade vinyl. The primary outcome was serious fall-related injury over 4 years that required an emergency department visit or hospital admission and a treatment procedure or diagnostic evaluation in hospital. Secondary outcomes included minor fall-related injury, any fall-related injury, falls, and fracture. Outcomes were ascertained by blinded assessors between September 1, 2013 and August 31, 2017 and analyzed by intention to treat. Adverse outcomes were not assessed. During follow-up, 184 residents occupied 74 intervention rooms, and 173 residents occupied 76 control rooms. Residents were 64.3% female with mean (SD) baseline age 81.7 (9.5) years (range 51.1 to 104.6 years), body mass index 25.9 (7.7) kg/m^2^, and follow-up 1.64 (1.39) years. 1,907 falls were reported; 23 intervention residents experienced 38 serious injuries (from 29 falls in 22 rooms), while 23 control residents experienced 47 serious injuries (from 34 falls in 23 rooms). Compliant flooring did not affect odds of ≥1 serious fall-related injury (12.5% intervention versus 13.3% control, odds ratio [OR]: 0.98, 95% CI: 0.52 to 1.84, *p* = 0.950) or ≥2 serious fall-related injuries (5.4% versus 7.5%, OR: 0.74, 95% CI: 0.31 to 1.75, *p* = 0.500). Compliant flooring did not affect rate of serious fall-related injuries (0.362 versus 0.422 per 1,000 bed nights, rate ratio [RR]: 1.04, 95% CI: 0.45 to 2.39, *p* = 0.925; 0.038 versus 0.053 per fall, RR: 0.81, 95% CI: 0.38 to 1.71, *p* = 0.560), rate of falls with ≥1 serious fall-related injury (0.276 versus 0.303 per 1,000 bed nights, RR: 0.97, 95% CI: 0.52 to 1.79, *p* = 0.920), or time to first serious fall-related injury (0.237 versus 0.257, hazard ratio [HR]: 0.92, 95% CI: 0.52 to 1.62, *p* = 0.760). Compliant flooring did not affect any secondary outcome in this study. Study limitations included the following: findings were specific to 2.54 cm SmartCells compliant flooring installed in LTC resident rooms, standard fall and injury prevention interventions were in use throughout the study and may have influenced the observed effect of compliant flooring, and challenges with concussion detection in LTC residents may have prevented estimation of the effect of compliant flooring on fall-related concussions.

**Conclusions:**

In contrast to results from previous retrospective and nonrandomized studies, this study found that compliant flooring underneath hospital-grade vinyl was not effective at preventing serious fall-related injuries in LTC. Future studies are needed to identify effective methods for preventing fall-related injuries in LTC.

**Trial registration:**

ClinicalTrials.gov: NCT01618786

## Abstract 2

Dawn Mackey and co-workers trial low-stiffness flooring in prevention of fall-related injuries among older adults in long-term care.

## Author summary

**Why was this study done?:**

- Compliant (low-stiffness) flooring aims to reduce impact forces on the body and thereby prevent injuries from falls.
- Controlled laboratory studies have shown that specific types of compliant flooring provide meaningful amounts of force attenuation and energy absorption during impact to vulnerable locations, such as the hip and head, without impairing balance or mobility during daily activities, such as standing and walking.
- Preliminary evidence suggested compliant flooring may be effective at preventing fall-related injuries in long-term care, but past studies were limited by nonrandomized or retrospective designs and insufficient sample sizes; no randomized trial has tested the effectiveness of compliant flooring at preventing serious fall-related injuries in long-term care.

**What did the researchers do and find?:**

- To our knowledge, we conducted the first randomized trial of compliant flooring in long-term care (FLIP Study). We believe it is the largest and most methodologically rigorous study of compliant flooring conducted to date.
- The compliant flooring we studied (SmartCells 2.54 cm underneath 2 mm hospital-grade vinyl) did not reduce the frequency of serious fall-related injuries, minor fall-related injuries, or falls among long-term care residents over 4 years.

**What do these findings mean?:**

- Toward the design of safer living environments for older adults, our study demonstrates that installation of the compliant flooring we studied would not increase the risk or rate of falls among long-term care residents, but it would also not reduce the risk or rate of fall-related injury.
- The compliant flooring we investigated is not likely to cause harm to long-term care residents, but we were unable to detect benefits to residents based on the metrics we examined.
- The results of this study may inform policies, programs, and practices for fall injury prevention in long-term care, but future research is needed to identify effective methods for reducing morbidity from falls.

## Introduction

Falls are a major health concern for older adults world-wide, particularly in long-term care (LTC), where approximately 60% of residents fall at least once per year, and 30% of falls cause injury [1–3]. Despite implementation of evidence-informed fall prevention programs, preventing falls in LTC remains a significant challenge [4–6] because residents present with complex medical histories and multiple risk factors. Vitamin D supplementation leads to modest reductions in fall rates [4]. Multifactorial fall prevention programs may reduce risk of falling, but implementation is challenging, time-consuming, and costly because they are delivered by multidisciplinary teams and customized to individual risks [5]. Given persistently high rates of falls in LTC, complementary solutions are needed, and healthcare stakeholders are increasingly considering the adoption of technologies to prevent injuries when falls happen.

Compliant flooring is a technology that aims to decrease the stiffness of the ground surface and the subsequent forces applied to the body during fall impact [7]. Extensive biomechanical research demonstrates that specific types of compliant flooring provide substantial impact force attenuation without impairing balance or mobility during daily activities [8–13]. Preliminary evidence suggests compliant flooring may be effective at preventing fall-related injuries in LTC [14–16]. Past studies have been limited, however, by retrospective and/or nonrandomized designs and insufficient sample sizes to examine the effect of compliant flooring on the most serious and costly fall-related injuries. LTC stakeholders and injury prevention researchers have called for evidence on the clinical effectiveness of compliant flooring from randomized trials [15,17,18].

Our goal was to address this evidence gap by conducting a randomized trial of compliant flooring in LTC. Our primary objective was to assess the clinical effectiveness of compliant flooring at preventing serious fall-related injuries among residents of LTC, relative to control flooring. Our secondary objectives were to assess the clinical effectiveness of compliant flooring at preventing minor fall-related injuries, all fall-related injuries, fractures, and falls among residents of LTC relative to control flooring.

## Methods

### Study design

The Flooring for Injury Prevention (FLIP) Study was a 4-year, parallel-group, two-arm, randomized superiority trial of flooring in 150 single-occupancy resident rooms at a single LTC site in British Columbia, Canada (New Vista Care Home). The trial began in September, 2013 and ended in August, 2017, as planned. The study was approved by the research ethics boards at Simon Fraser University (2013s0535) and the Fraser Health Authority (2012–059). The study protocol was published previously and included a prospective analysis plan (S1 Protocol) [19]. Reporting was guided by the CONSORT checklist (S1 Checklist) [20].

### Rooms and residents

**Fig 1:**
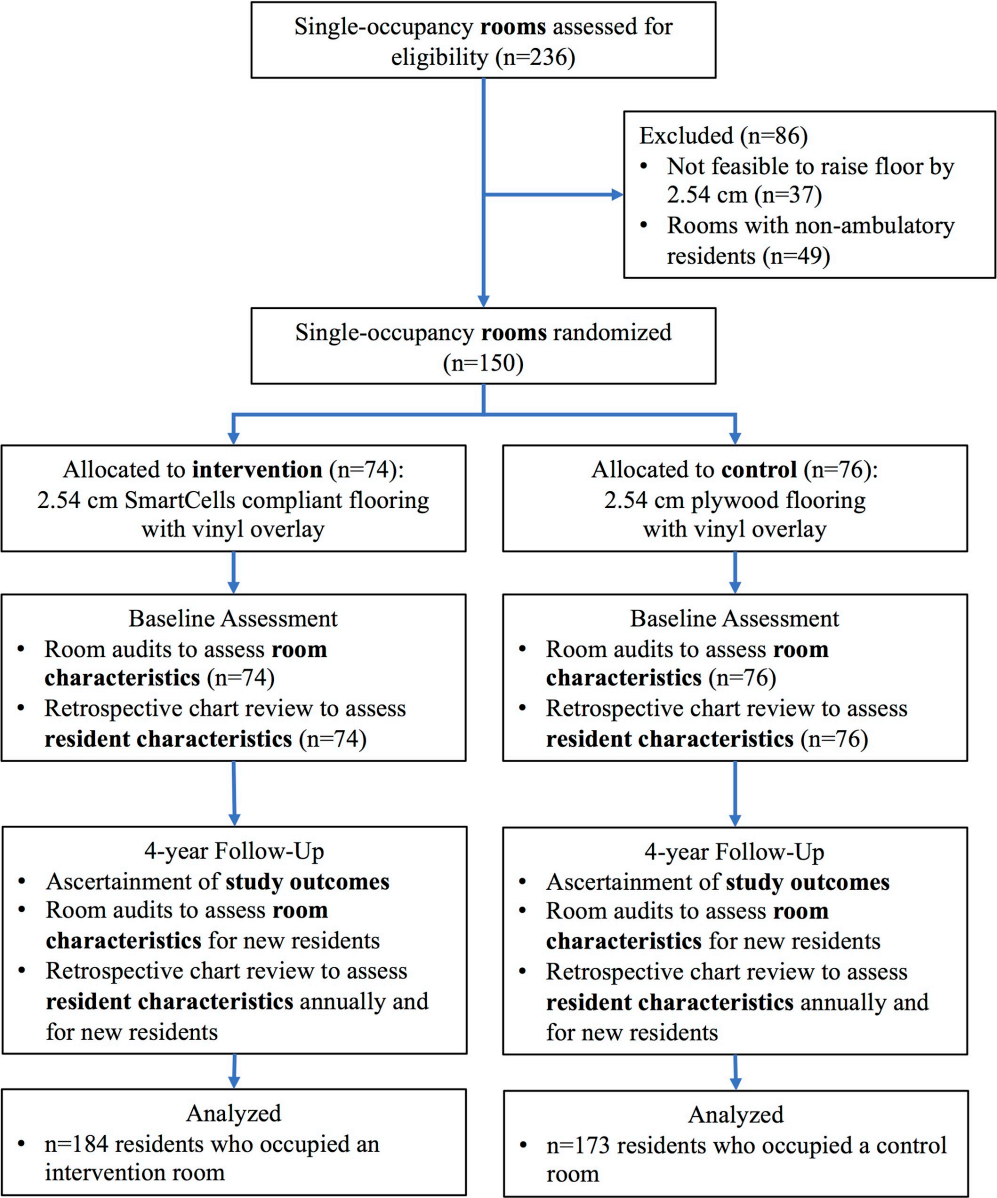
Trial profile for the FLIP Study, 2013–2017. FLIP, Flooring for Injury Prevention.

The LTC site had 236 resident rooms that were located within five residential villages (units). Of the 236 resident rooms, 150 rooms from four of the residential villages were included **(**Fig 1**)**. Rooms were ineligible if the existing floor could not accommodate installation of the intervention and control floors (n = 37). Resident rooms on the third floor (the fifth residential village) (n = 49) were also ineligible because the majority of residents primarily used a wheelchair for their mobility and therefore carried a different baseline risk for falls and injuries. As described previously, informed consent was not obtained from residents of study rooms [19]. This was appropriate because the LTC site undertook an environmental-level intervention of their resident rooms (i.e., installation of new flooring) rather than an individual-level intervention, and the study’s data collection activities were retrospective and involved only secondary and deidentified data.

### Randomization and masking

Study rooms, and residents living within those rooms, were randomized before flooring installation began. Randomization was stratified by residential village (4 villages total) in blocks of 4 rooms with 1:1 allocation. Randomization was performed by the principal investigator using a computerized random-number generator and concealed from residents, residents’ families, LTC staff (outcome assessors), and FLIP Study research assistants involved in data collection and analysis. The sequence was shared immediately after generation with the flooring installation team and an on-site project manager at the LTC site to enable room renovations; neither party was involved in data collection or analysis. During follow-up, LTC staff assigned incoming residents to rooms on a first come, first served basis, which was mandatory practice; flooring was not considered when making room assignments. We considered this process quasirandom assignment, and it served to maintain masking of LTC staff.

Intervention and control flooring were covered with the same hospital-grade vinyl, and thus, intervention and control rooms looked identical. We took specific actions to protect against potential bias that could arise from perceived differences in floor stiffness: (i) the flooring installation team, LTC residents and their families, and LTC staff were unaware of the specific research questions throughout the trial; and (ii) LTC residents, their families, and LTC staff were also unaware of the types or numbers of floors under evaluation to minimize the chance that reporting of fall-related injuries would be influenced by any real or perceived knowledge of the flooring on which falls occurred.

### Procedures

#### Intervention: Compliant flooring

The intervention compliant flooring was SmartCells (2.54 cm, SATECH, Inc., Chehalis, WA, US). It was installed over existing concrete floors covering the living, bathroom, and closet areas of 74 resident rooms. SmartCells is a synthetic rubber floor (surface hardness 50 durometer, density 1,120 kg/m^3^) comprised of a continuous surface layer supported underneath by an array of cylindrical rubber columns (14 mm in diameter, spaced at 19 mm intervals). SmartCells was covered with hospital-grade vinyl (2.0 mm AltroSmooth Ethos, Altro, Mississauga, ON, Canada).

#### Control: Plywood flooring

The control flooring was plywood (2.54 cm). It was installed over existing concrete floors covering the living, bathroom, and closet areas of 76 resident rooms and in hallways adjacent to resident rooms. Plywood served as a rigid floor because it provides minimal force attenuation (1.6%) versus concrete during impacts from simulated falls on the hip [21,22]; plywood was covered with the same hospital-grade vinyl as the SmartCells compliant flooring.

### Outcomes

#### Primary outcome

Serious fall-related injury was the primary outcome. It was defined as any impact-related injury due to a fall in a study room that (i) resulted in an emergency department visit or a hospital admission and (ii) met one of the following criteria: fractures had to be confirmed via X-ray; lacerations had to be sutured; and a treatment procedure or a diagnostic evaluation had to be performed for hematomas, sprains/strains, contusions/bruises, swelling, pain, abrasions, and other injury types. If these criteria were not met, the injury was considered a minor fall-related injury. Injuries from falls in common areas (e.g., dining rooms, hallways, lounges) were not considered.

#### Secondary outcomes

The FLIP Study included four secondary outcomes: (1) minor fall-related injury, defined as any impact-related injury due to a fall in a study room that did not meet criteria for a serious fall-related injury; (2) any fall-related injury (minor or serious); (3) fall, defined as an unexpected event in which a resident came to rest on the ground, floor, or lower level in a study room [23]; and (4) fracture due to a fall in a study room.

#### Safety outcomes and adverse events

We did not define or track any safety outcomes or adverse events.

#### Measurement of study outcomes

Study outcomes were monitored at the LTC site from September 1, 2013 through August 31, 2017. According to standard practice, nursing staff at the LTC site completed an incident form within 24 hours of each resident fall. Trained study research assistants reviewed incident forms to ascertain the date, time, and location of each fall and details about the nature and extent of any injuries apparent at the time of the fall. They also ascertained whether an emergency department visit and/or hospital admission occurred because of a fall-related injury. Further, they reviewed resident charts for 7 days postfall and hospital records (if applicable) to ascertain additional details about fall-related injuries. Together, information from incident forms, resident charts, and hospital records were used to assess the nature and extent of fall-related injuries, including injury type, location, and severity.

### Other measures

#### Resident characteristics

We ascertained resident-level characteristics that may influence risk for falls and injuries from retrospective reviews of the Minimum Data Set (MDS 2.0, interRAI Corporation 1999, Rockport, MA, US), an observational assessment of a resident’s medical, functional, psychological, and cognitive status that British Columbia mandates is completed annually and updated quarterly for all LTC residents. These included age; sex; body mass index; medical conditions; fall and fracture history; use of a cane, walker, wheelchair, or transfer aid; unsteady gait; ability to maintain standing balance independently; and medications. We ascertained characteristics of all residents at trial baseline; during follow-up, we ascertained characteristics annually and upon new resident admissions. Data were doubly entered by trained research assistants; regular comparisons were performed to identify discrepancies and correct errors.

#### Room characteristics

We ascertained room-level characteristics that might influence risk for falls and injuries by performing room audits. We assessed square footage of the whole room and bathroom; wheelchair accessibility of the bathroom; and presence of a bathroom grab bar, ceiling lift, and fall mat. Room audits were performed in all study rooms after flooring installation was complete and before outcome monitoring started. Audits were also performed during follow-up when room occupancy changed (e.g., new resident admission, internal move of existing resident). Data were doubly entered by trained research assistants; regular comparisons were performed to identify discrepancies and correct errors.

### Statistical analysis

The FLIP Study was designed to detect a 35%–40% reduction in the frequency of serious fall-related injuries between groups with two-sided significance level of 0.05 and power of 80% [19]. We projected a 1-year cumulative incidence of serious fall-related injury in the control arm of 16.6% (4-year cumulative incidence of 66.2%). Interim analysis was not planned, and stopping guidelines were not specified since the intervention involved an environmental modification. An independent three-member data and safety monitoring board with expertise in biostatistics, clinical trial design, and LTC met three times to review the study’s progress, including blinded tables of overall outcome frequencies.

Baseline characteristics of residents and rooms according to group assignment were reported as mean (SD) for normally distributed continuous variables, median (IQR) for skewed continuous variables, and n (%) for categorical variables. Incidence rates of study outcomes were calculated as marginal rates (total number of events/sum of person time).

For each outcome, we summarized the data using a set of outcome measures consistent with international guidelines by the Prevention of Falls Network Europe [23]. This was an update from our published protocol, intended to strengthen our reporting and decided before we analyzed the data; specific outcome measures are defined below. We conducted intent-to-treat analyses using a two-tailed significance level of 0.05. Analyses were performed using R, version 3.4.1 (RStudio version 1.0.153, Inc., Boston, MA, US). The trial was registered with ClinicalTrials.gov (NCT01618786).

For each outcome measure, we fitted a base model with minimal (or no) covariate adjustment and a multivariable adjusted model. To select covariates for multivariable models, we identified baseline covariates that appeared to be meaningfully imbalanced between groups or were known from past research to be strongly associated with the study outcomes. These included age (<85, ≥85 years), body mass index, cognitive function by Mini Mental State Examination, history of ≥1 falls in past 180 days, inability to stand independently, diagnosis of dementia, use of antianxiety or analgesic medication, wheelchair as primary mode of transportation, and presence of fall mat and ceiling lift in resident room. Body mass index and cognitive function were dropped from further consideration because of excessive missing data. Next, we dropped covariates that were not associated at *p* < 0.05 with at least one of the following study outcome measures: rate of serious fall-related injuries, rate of minor fall-related injuries, or rate of falls. We verified lack of co-linearity among remaining covariates. Factors in the final multivariable models included age, history of ≥1 falls in past 180 days, inability to stand independently, diagnosis of dementia, antianxiety medication, and analgesic medication.

#### Serious fall-related injury outcomes

We conducted binary logistic regression analysis to examine intervention effects on (i) ≥1 serious fall-related injuries and (ii) ≥2 serious fall-related injuries. We conducted negative binomial regression analysis to examine intervention effects on (i) number of serious fall-related injuries per 1,000 bed nights (offset = exposure time in 1,000 bed nights), (ii) number of serious fall-related injuries per fall (offset = number of falls, subset to residents who had ≥1 falls and therefore not a randomized comparison), and (iii) number of falls that resulted in at least one serious fall-related injury (offset = exposure time in 1,000 bed nights). We used negative binomial regression models rather than Poisson models, as specified in our prospective analysis plan (S1 Protocol), because they better fit the distribution of the data. Finally, we examined the intervention effect on time to first serious fall-related injury with Cox proportional hazards models. Base binary logistic regression models were adjusted only for exposure time (bed nights); all other base models were unadjusted.

#### Minor and any fall-related injury outcomes

We conducted the same analyses as described for serious fall-related injuries.

#### Fall outcomes

We conducted binary logistic regression analysis to examine intervention effects on (i) ≥1 falls and (ii) ≥2 (recurrent) falls. We conducted negative binomial regression analysis to examine intervention effects on number of falls per 1,000 bed nights (offset = exposure time in 1,000 bed nights). We examined intervention effects on time to first fall with a Cox proportional hazards model. Base binary logistic regression models were adjusted only for exposure time (bed nights); all other base models were unadjusted.

#### Fractures

We did not fit regression models for fractures because the number of fractures was insufficient.

#### Subgroups

We evaluated the effectiveness of compliant flooring on serious fall-related injury outcomes in six subgroups, chosen based on evidence documenting differences in fall-related injury risk and/or hypothesized differences in response to compliant flooring: age (<85, 85+ years), sex, BMI (≤25, >25 kg/m^2^), dementia, ≥1 falls in past 80 days, and ability to maintain standing balance independently. Subgroup analyses were conducted by testing for significant treatment by covariate interactions at *p* < 0.05. Subgroup analyses were not specified in our prospective analysis plan (S1 Protocol), but they were conducted as exploratory analyses.

#### Sensitivity analyses

To explore the influence of residents who fell frequently, residents who had fall rates in the top fifth percentile (n = 21) were excluded, and analyses were rerun.

## Results

Over 4 years follow-up, 173 residents occupied control rooms, and 184 residents occupied intervention rooms for ≥1 bed night. Of these, 19 moved internally within the site from an intervention to control room, and 13 others moved from a control to intervention room, resulting in 325 unique residents who occupied the 150 study rooms. Decisions to move a resident internally were made by LTC managers and staff when changes in resident health status led to different care needs (e.g., need for ceiling lift, wheelchair-accessible bathroom, dementia unit, proximity to spouse). During follow-up, there were 42 deaths among residents who occupied control rooms and 48 deaths among residents who occupied intervention rooms.

At baseline, residents had a mean (SD) age of 81.7 (9.5) years; 64.3% were female **(**Table 1**)**. Baseline resident characteristics were well balanced between intervention and control groups across a range of demographic, medical history, mobility, and medication variables, with some exceptions. The intervention group was more likely to have diagnoses of dementia and depression, a history of falls in the past 180 days, and inability to stand independently. The intervention group was more likely to be prescribed antianxiety medication and less likely to be prescribed analgesic medication. Mean (SD) follow-up time among residents was 1.64 (1.39) years (range: 1 day to 4 years); mean follow-up time was 1.56 (1.37) years in the intervention group and 1.72 (1.41) years in the control group. Despite resident turnover, resident characteristics remained well balanced over time between groups, apart from exceptions noted above.

**Table 1:** Baseline characteristics of residents and rooms in the FLIP Study.

	Overall	Compliant Flooring (INT)	Control Flooring (CON)
**Resident Characteristics**	(n = 357)	(n = 184)	(n = 173)
**Demographic**			
Age, years	81.7 (9.5)	81.2 (9.9)	82.1 (9.1)
<85	206 (57.7)	108 (58.7)	98 (56.6)
≥85	151 (42.3)	76 (41.3)	75 (43.4)
Women	229 (64.3)	119 (64.7)	110 (64.0)
BMI, kg/m^2^	25.9 (7.7)	26.3 (8.3)	25.4 (7.0)
<25	186 (54.4)	92 (51.4)	94 (57.7)
≥25	156 (45.6)	87 (48.6)	69 (42.3)
Village			
Village A	50 (14.0)	25 (13.6)	25 (14.5)
Village B	48 (13.5)	27 (14.7)	21 (12.1)
Village C	66 (18.5)	33 (17.9)	33 (19.1)
Village D	193 (54.1)	99 (53.8)	94 (54.3)
Do not hospitalize directive	180 (56.4)	94 (56.6)	86 (56.2)
**Medical Conditions**			
Visual impairment	106 (29.7)	54 (29.4)	52 (30.1)
CVD	32 (9.0)	18 (9.8)	14 (8.1)
Hypertension	135 (37.8)	64 (34.8)	71 (41.0)
Stroke or TIA	32 (9.0)	18 (9.8)	14 (8.1)
Arthritis	83 (23.3)	39 (21.2)	44 (25.6)
Osteoporosis	44 (12.3)	23 (12.5)	21 (12.1)
Alzheimer's disease	56 (15.7)	29 (15.8)	27 (15.6)
Dementia	196 (54.9)	104 (56.5)	92 (53.2)
Depression	46 (12.9)	27 (14.7)	19 (11.0)
Parkinson's disease	16 (4.5)	7 (3.8)	9 (5.2)
≥1 falls, past 180 days	75 (21.0)	41 (22.3)	34 (19.7)
Hip fracture, past 180 days	5 (1.4)	2 (1.1)	3 (1.7)
Other fracture, past 180 days	5 (1.4)	3 (1.6)	2 (1.2)
Number of medical conditions	1.8 (1.2)	1.8 (1.3)	1.8 (1.2)
**Mobility**			
Short-form ADL complete dependence	8 (2.2)	5 (2.7)	3 (1.7)
Unable to stand independently	75 (21.0)	42 (22.8)	33 (19.1)
Use of cane, walker, or crutch	145 (40.6)	75 (40.8)	70 (40.5)
Wheelchair primary mode of transportation	54 (15.1)	29 (15.8)	25 (14.5)
Use of transfer aid	28 (7.8)	15 (8.2)	13 (7.5)
Unsteady gait	58 (16.3)	29 (15.8)	29 (16.8)
**Medications, Past 7 Days**			
Number of medications	8.1 (3.8)	8.2 (3.7)	7.9 (4.0)
Antipsychotic	133 (37.3)	71 (38.6)	62 (35.8)
Antianxiety	58 (16.3)	38 (20.7)	20 (11.6)
Antidepressant	159 (44.5)	87 (47.3)	72 (41.6)
Hypnotic	86 (24.1)	45 (24.5)	41 (23.7)
Diuretic	80 (22.4)	39 (21.2)	41 (23.7)
Analgesic	196 (54.9)	94 (51.1)	102 (59.0)
**Room Characteristics**		(n = 74)	(n = 76)
Whole room area, square feet	167.2 (8.0)	167.6 (8.1)	166.9 (8.0)
Bathroom area, square feet*	20.4 (12.5)	20.3 (10.8)	20.5 (12.3)
Wheelchair accessible bathroom	186 (52.3)	91 (49.7)	95 (54.9)
≥1 grab bars in bathroom	206 (57.9)	103 (56.3)	103 (59.5)
Ceiling lift	30 (8.5)	11 (6.0)	19 (11.1)
Fall mat	65 (18.3)	30 (16.4)	35 (20.4)

Cells contain mean (SD) for continuous variables and n (%) for categorical variables, unless otherwise indicated. Number of chronic medical conditions includes count of CVD (includes atherosclerotic heart disease and cardiac dysrhythmia), hypertension, stroke or TIA, arthritis, osteoporosis, Alzheimer’s disease, dementia, depression, and Parkinson’s disease

*Median (interquartile range). BMI missing for 5 INT and 10 CON; do not hospitalize directive missing for 18 INT and 20 CON; arthritis missing for 1 CON; wheelchair accessible bathroom missing for 1 INT; 1+ grab bars in bathroom missing for 1 INT; ceiling lift missing for 1 INT and 1 CON; fall mat missing for 1 INT and 1 CON.

**Abbreviations:** ADL, activities of daily living; BMI, body mass index; CON, Control; CVD, Cardiovascular disease; FLIP, Flooring for Injury Prevention; INT, intervention; TIA, transient ischemic attack.

Fixed characteristics of resident rooms were comparable between intervention and control groups (Table 1), but fewer rooms in the intervention group had ceiling lifts or fall mats. Mean (SD) follow-up time among study rooms was 3.90 (0.14) years (range: 2.95 years to 4 years); study rooms were occasionally unoccupied because of overnight hospital and social leaves as well as during periods of resident turnover. Mean follow-up time was 3.89 (0.17) years for intervention rooms and 3.92 (0.11) years for control rooms.

### Primary outcome: Serious fall-related injury

During follow-up, 46 residents (14.1% of unique residents) experienced 85 serious fall-related injuries in 45 unique rooms from 63 falls; 47 falls caused a single serious fall-related injury, 11 falls caused 2 serious fall-related injuries, and 5 falls caused ≥3 serious fall-related injuries (Table 2). Of the 85 serious injuries, 38 occurred in the intervention group (from 29 falls), and 47 occurred in the control group (from 34 falls). The most common types of serious fall-related injuries were pain (35.3%), laceration/cut (22.4%), and fracture (21.2%) (Table 2). The most common locations of serious fall-related injuries were head/skull (21.2%), hip (14.1%), and spine/neck/pelvis (12.9%). Four hip injuries were fractures. The frequency of serious fall-related injuries appeared highest in the first and fourth years.

**Table 2:** Frequency of serious fall-related injuries in the FLIP Study, 2013–2017.

	Overall	Compliant Flooring (INT)	Control Flooring (CON)
	n (%)	n (%)	n (%)
Total number of serious injuries	85	38	47
Year of follow-up			
Year 1	24 (28.2)	7 (18.4)	17 (36.2)
Year 2	17 (20.0)	10 (26.3)	7 (14.9)
Year 3	19 (22.4)	10 (26.3)	9 (19.2)
Year 4	25 (29.4)	11 (29.0)	14 (29.8)
Type			
Pain	30 (35.3)	12 (31.6)	18 (38.3)
Laceration/cut	19 (22.4)	8 (21.1)	11 (23.4)
Fracture	18 (21.2)	8 (21.1)	10 (21.3)
Contusion/bruise	10 (11.8)	4 (10.5)	6 (12.8)
Hematoma	2 (2.4)	1 (2.6)	1 (2.1)
Sprain/strain	2 (2.4)	1 (2.6)	1 (2.1)
Swelling	2 (2.4)	2 (5.3)	0 (0.0)
Dislocation	1 (1.2)	1 (2.6)	0 (0.0)
Other	1 (1.2)	1 (2.6)	0 (0.0)
Location			
Head/skull	18 (21.2)	7 (18.4)	11 (23.4)
Face	4 (4.7)	2 (5.3)	2 (4.3)
Neck/spine/pelvis	11 (12.9)	5 (13.2)	6 (12.8)
Hip	12 (14.1)	4 (10.5)	8 (17.0)
Leg	9 (10.6)	2 (5.3)	7 (14.9)
Shoulder/clavicle/scapula	7 (8.2)	6 (15.8)	1 (2.1)
Arm	9 (10.6)	3 (7.9)	6 (12.8)
Wrist	1 (1.2)	0 (0.0)	1 (2.1)
Chest/ribs/sternum	8 (9.4)	5 (13.2)	3 (6.4)
Ankle/foot/heel/toes	3 (3.5)	2 (5.3)	1 (2.1)
Hand/fingers	3 (3.5)	2 (5.3)	1 (2.1)
Other*	0 (0)	0 (0)	0 (0)

*Other injury locations include abdomen and unspecified.

**Abbreviations:** CON, control; FLIP, Flooring for Injury Prevention; INT, intervention.

Relative to control, compliant flooring did not affect the odds of residents sustaining (i) ≥1 serious fall-related injuries (12.5% intervention versus 13.3% control, base model odds ratio [OR]: 0.98, 95% CI: 0.52 to 1.84, *p* = 0.950) or (ii) ≥2 serious fall-related injuries (5.4% versus 7.5%, base model OR: 0.74, 95% CI: 0.31 to 1.75, *p* = 0.500) (Table 3). Relative to control, compliant flooring also did not affect (i) the number of serious fall-related injuries per 1,000 bed nights (0.362 versus 0.422, base model rate ratio [RR]: 1.04, 95% CI: 0.45 to 2.39, *p* = 0.925), (ii) the number of serious fall-related injuries per fall (0.038 versus 0.053, base model RR: 0.81, 95% CI: 0.38 to 1.71, *p* = 0.560), (iii) the number of falls with at least one fall-related injury per 1,000 bed nights (0.276 versus 0.303, base model RR: 0.97, 95% CI: 0.52 to 1.79, *p* = 0.920), or (iv) time to first serious fall-related injury (0.237 versus 0.257, base model hazard ratio [HR]: 0.92, 95% CI: 0.52 to 1.62, *p* = 0.760). Results were unaltered by multivariable adjustment (Table 3) or exclusion of the most frequent fallers (S1 Table). All subgroup analyses were nonsignificant (S2 Table).

**Table 3:** Comparison of serious fall-related injuries between compliant flooring INT and control flooring CON groups in the FLIP Study, 2013–2017.

	Compliant Flooring		Control Flooring		Base Model^a^		Multivariable Model^b^	
	INT		CON					
	(n = 184)		(n = 173)					
Serious Fall-Related Injury	Events	Risk	Events	Risk	OR (95% CI)	*p*	OR (95% CI)	*p*
≥1 serious fall-related injury	23	12.5	23	13.3	0.98 (0.52, 1.84)	0.950	0.99 (0.52, 1.92)	0.977
≥2 serious fall-related injuries	10	5.4	13	7.5	0.74 (0.31, 1.75)	0.500	0.78 (0.31, 1.95)	0.594
	Events	Rate	Events	Rate	RR (95% CI)	*p*	RR (95% CI)	*p*
Number of serious fall-related injuries/1,000 bed nights	38	0.362	46	0.422	1.04 (0.45, 2.39)	0.925	1.23 (0.55, 2.76)	0.603
Number of serious fall-related injuries/fall	38	0.038	46	0.053	0.81 (0.38, 1.71)	0.560	0.90 (0.42, 1.92)	0.769
Number of falls with ≥1 serious fall-related injury/1,000 bed nights	29	0.276	33	0.303	0.97 (0.52, 1.79)	0.920	1.02 (0.55,1.88)	0.955
	Events	Rate	Events	Rate	HR (95% CI)	*p*	HR (95% CI)	*p*
Time to first serious fall-related injury^c^	23	0.237	24	0.257	0.92 (0.52,1.62)	0.760	0.91 (0.50, 1.64)	0.727

^a^Includes main effect term for intervention group (1 = INT, 0 = CON). For binary logistic models that generated ORs, bed nights of follow-up was a covariate. For negative binomial models that generated RRs, offset was specified as bed nights of follow-up for endpoint of number of serious fall-related injuries/1,000 bed nights, and offset was specified as number of falls for endpoint of number of serious fall-related injuries/fall.

^b^Base model plus adjustment for baseline values for the following five covariates: age (<85, 85+ years), dementia, ≥1 fall in the past 180 days, antianxiety medication, and analgesic medication.

^c^Rate expressed as events per 1,000 bed nights.

**Abbreviations:** CON, control; FLIP, Flooring for Injury Prevention; HR, hazard ratio; INT, intervention; OR, odds ratio; RR, rate ratio.

### Secondary outcomes: Minor fall-related injury, any fall-related injury, and falls

During follow-up, 162 residents (49.8% of unique residents) experienced 732 minor fall-related injuries in 123 unique rooms from 530 falls; 382 falls caused a single minor fall-related injury, 111 falls caused 2 minor fall-related injuries, and 37 falls caused ≥3 minor fall-related injuries (Table 4). Of the 732 minor injuries, 358 occurred in the intervention group (from 247 falls), and 374 occurred in the control group (from 283 falls). The most common types of minor injuries were pain (44.4%), contusion/bruise (19.0%), and laceration/cut (17.8%). The most common locations of minor fall-related injuries were spine/neck/pelvis (20.2%), leg (16.3%), and arm (16.1%). The frequency of minor fall-related injuries tended to be greater in the first year.

**Table 4:** Frequency of minor fall-related injuries in the FLIP Study, 2013–2017.

	Overall	Compliant Flooring	Control Flooring
		(INT)	(CON)
	n (%)	n (%)	n (%)
Total number of minor injuries	732	358	374
Year of follow-up			
Year 1	206 (28.1)	104 (29.1)	102 (27.3)
Year 2	165 (22.5)	77 (21.5)	88 (23.5)
Year 3	184 (25.1)	97 (27.1)	87 (23.3)
Year 4	177 (24.2)	80 (22.3)	97 (25.9)
Type			
Pain	325 (44.4)	147 (41.1)	178 (47.6)
Contusion/bruise	139 (19.0)	74 (20.7)	65 (17.4)
Laceration/cut	130 (17.8)	68 (19.0)	62 (16.6)
Abrasion	69 (9.4)	38 (10.6)	31 (8.3)
Hematoma	49 (6.7)	16 (4.5)	33 (8.8)
Swelling	18 (2.5)	14 (3.9)	4 (1.1)
Sprain/strain	1 (0.1)	0 (0.0)	1 (0.3)
Other	1 (0.1)	1 (0.3)	0 (0.0)
Location			
Head/skull	101 (13.8)	43 (12.0)	58 (15.5)
Face	43 (5.9)	18 (5.0)	25 (6.7)
Neck/spine/pelvis	148 (20.2)	71 (19.8)	77 (20.6)
Hip	28 (3.8)	12 (3.4)	16 (4.3)
Leg	119 (16.3)	59 (16.5)	60 (16.0)
Shoulder/clavicle/scapula	39 (5.3)	18 (5.0)	21 (5.6)
Arm	118 (16.1)	60 (16.8)	58 (15.5)
Wrist	16 (2.2)	8 (2.2)	8 (2.1)
Chest/ribs/sternum	17 (2.3)	10 (2.8)	7 (1.9)
Ankle/foot/heel/toes	26 (3.6)	17 (4.8)	9 (2.4)
Hand/fingers	42 (5.7)	23 (6.4)	19 (5.1)
Other*	35 (4.8)	19 (5.3)	16 (4.3)

*Other injury locations include abdomen and unspecified.

**Abbreviations:** CON, control; FLIP, Flooring for Injury Prevention; INT, intervention.

Relative to control, compliant flooring did not affect the risk of residents sustaining (i) ≥1 minor (or any) fall-related injuries or (ii) ≥2 minor (or any) fall-related injuries (Table 5). Relative to control, compliant flooring also did not affect (i) the number of minor (or any) fall-related injuries per 1,000 bed nights, (ii) the number of minor (or any) fall-related injuries per fall, (iii) the number of falls with at least one minor (or any) fall-related injury per 1,000 bed nights, or (iv) time to first minor (or any) fall-related injury. Results were unaltered by multivariable adjustment (Table 5) or exclusion of the most frequent fallers (S3 Table).

**Table 5:** Comparison of secondary outcomes between compliant flooring INT and control flooring CON groups in the FLIP Study, 2013–2017.

	Compliant Flooring		Control Flooring		Base Model^a^		Multivariable Model^b^	
	INT		CON					
	(n = 184)		(n = 173)					
Minor Fall-Related Injury	Events	Risk	Events	Risk	OR (95% CI)	*p*	OR (95% CI)	*p*
≥1 minor fall-related injury	87	47.3	78	45.1	1.19 (0.77, 1.84)	0.450	1.36 (0.86, 2.18)	0.193
≥2 minor fall-related injuries	63	34.2	58	33.5	1.12 (0.71, 1.77)	0.630	1.28 (0.79, 2.10)	0.315
	Events	Rate	Events	Rate	RR (95% CI)	*p*	RR (95% CI)	*p*
Number of minor fall-related injuries/1,000 bed nights	353	3.363	362	3.325	0.96 (0.64, 1.46)	0.850	1.24 (0.83, 1.87)	0.280
Number of minor fall-related injuries/fall	353	0.357	362	0.414	1.01 (0.79, 1.29)	0.940	1.14 (0.88, 1.47)	0.338
Number of falls with ≥1 minor fall-related injury/1,000 bed nights	243	2.315	275	2.526	0.91 (0.62, 1.34)	0.610	1.11 (0.76, 1.63)	0.594
	Events	Rate	Events	Rate	HR (95% CI)	*p*	HR (95% CI)	*p*
Time to first minor fall-related injury^c^	87	1.32	79	1.24	1.04 (0.77, 1.42)	0.780	1.08 (0.79, 1.48)	0.645
**Any Fall-Related Injury**	Events	Risk	Events	Risk	OR (95% CI)	*p*	OR (95% CI)	*p*
≥1 fall-related injury	91	49.5	85	49.1	1.08 (0.70, 1.67)	0.715	1.21 (0.76, 1.91)	0.425
≥2 fall-related injuries	66	35.9	63	36.4	1.06 (0.67, 1.67)	0.810	1.20 (0.74, 1.96)	0.471
	Events	Rate	Events	Rate	RR (95% CI)	*p*	RR (95% CI)	*p*
Number of fall-related injuries/1,000 bed nights	391	3.725	408	3.747	0.98 (0.65, 1.48)	0.920	1.26 (0.85, 1.88)	0.234
Number of fall-related injuries/fall	391	0.395	408	0.466	1.03 (0.80, 1.33)	0.830	1.11 (0.86, 1.43)	0.422
Number of falls with ≥ 1 fall-related injury/1,000 bed nights	256	2.439	296	2.719	0.91 (0.62, 1.32)	0.600	1.09 (0.75, 1.59)	0.637
	Events	Rate	Events	Rate	HR (95% CI)	*p*	HR (95% CI)	*p*
Time to first fall-related injury^c^	93	1.43	87	1.44	0.97 (0.72, 1.30)	0.840	0.96 (0.71, 1.31)	0.815
**Falls**	Events	Risk	Events	Risk	OR (95% CI)	*p*	OR (95% CI)	*p*
≥1 fall	128	69.9	117	67.9	1.18 (0.74, 1.89)	0.480	1.31 (0.80, 2.15)	0.288
≥2 falls	94	51.1	93	53.8	0.96 (0.62, 1.48)	0.846	1.00 (0.63, 1.58)	0.991
	Events	Rate	Events	Rate	RR (95% CI)	*p*	RR (95% CI)	*p*
Number of falls/1,000 bed nights	989	9.421	875	8.036	1.21 (0.87, 1.68)	0.250	1.32 (0.94, 1.84)	0.091
	Events	Rate	Events	Rate	HR (95% CI)	*p*	HR (95% CI)	*p*
Time to first fall^c^	126	2.64	116	2.51	1.03 (0.80, 1.33)	0.810	0.98 (0.76, 1.28)	0.906

^a^Includes main effect term for intervention group (1 = INT, 0 = CON). For binary logistic models that generated ORs, bed nights of follow-up was a covariate. For negative binomial models that generated RRs, offset was specified as bed nights of follow-up for endpoint of number of serious fall-related injuries/1,000 bed nights, and offset was specified as number of falls for endpoint of number of serious fall-related injuries/fall.

^b^Base model plus adjustment for baseline values for the following five covariates: age (<85, 85+ years), dementia, ≥1 fall in the past 180 days, antianxiety medication, and analgesic medication.

^c^Rate expressed as events per 1,000 bed nights.

**Abbreviations:** CON, control; FLIP, Flooring for Injury Prevention; HR, hazard ratio; INT, intervention; OR, odds ratio; RR, rate ratio.

During follow-up, 235 residents (72.3% of unique residents) experienced a total of 1,907 falls in 143 unique rooms (Table 6). Ninety-five percent of falls were unwitnessed. Fall frequency tended to be greater in the first year, and most falls occurred in the evening/night (30.7%), while the fewest occurred in the afternoon (17.1%). Most falls (86.2%) occurred in areas of the resident room other than the bathroom. Hip protectors were documented to be worn in 41.1% of falls, but data on hip protector use were missing for a large proportion of falls (40.3%).

**Table 6:** Frequencies and characteristics of falls in the FLIP Study, 2013–2017.

	Overall	Compliant Flooring (INT)	Control Flooring (CON)
	n (%)	n (%)	n (%)
Falls	1,907	1,009	898
Witnessed			
Yes	90 (4.8)	51 (5.1)	49 (5.5)
No	1,804 (95.2)	961 (95.9)	843 (94.5)
Year of follow-up			
Year 1	569 (29.8)	344 (34.1)	225 (25.1)
Year 2	418 (21.9)	232 (23.0)	186 (20.7)
Year 3	458 (24.0)	235 (23.3)	223 (24.8)
Year 4	462 (24.2)	198 (19.6)	264 (29.4)
Time of day			
Morning (6:00 AM–11:59 AM)	441 (26.6)	246 (27.1)	195 (25.9)
Afternoon (12:00 PM–5:59 PM)	284 (17.1)	143 (15.8)	141 (18.7)
Evening (6:00 PM–11:59 PM)	510 (30.7)	281 (31.0)	229 (30.4)
Overnight (12:00 AM–5:59 AM)	425 (25.6)	237 (26.1)	188 (25.0)
Location of fall			
Fall in resident room, not bathroom	1,643 (86.2)	874 (86.6)	769 (85.6)
Fall in resident room, in bathroom	221 (11.6)	115 (11.4)	106 (11.8)
Fall in co-resident’s room	43 (2.3)	20 (2.0)	23 (2.6)
Hip protector worn at time of fall			
Yes	783 (41.1)	430 (42.6)	353 (39.3)
No	356 (18.7)	190 (18.8)	166 (18.5)
Do not know/missing	768 (40.3)	389 (38.6)	379 (42.2)

Data were missing as follows: witnessed (n = 7 INT, n = 6 CON), time of fall (n = 102 INT, n = 145 CON), hip protector worn at time of fall (n = 389 INT, n = 379 CON).

**Abbreviations:** CON, control; FLIP, Flooring for Injury Prevention; INT, intervention.

Relative to control, compliant flooring did not affect the risk of residents sustaining (i) ≥1 falls (69.9% intervention versus 67.9% control, base model OR: 1.18, 95% CI: 0.74 to 1.89, *p* = 0.470) or (ii) ≥2 (recurrent) falls (51.1% versus 53.8%, base model OR: 0.96, 95% CI: 0.62 to 1.48, *p* = 0.846) (Table 5). Relative to control flooring, compliant flooring did not affect (i) the number of falls per 1,000 bed nights (9.421 versus 8.036, base model RR: 1.21, 95% CI: 0.87 to 1.68, *p* = 0.250), or (ii) time to first fall (2.64 versus 2.51, base model HR: 1.03, 95% CI: 0.80 to 1.33, *p* = 0.810). Results were unaltered by multivariable adjustment (Table 5) or exclusion of the most frequent fallers (S3 Table).

## Discussion

Over 4 years, compliant flooring (2.54 cm SmartCells covered with 2 mm hospital-grade vinyl) did not reduce the risk of serious fall-related injury, rate of serious fall-related injury (per 1,000 bed nights or per fall), rate of falls with at least one serious fall-related injury, or time to first serious fall-related injury among older adult residents in LTC. Our findings did not suggest that the compliant flooring used in this study had any effect on serious fall-related injury in any subgroup of residents. The compliant flooring used in this study also had no effect on secondary outcomes, including minor fall-related injury, any fall-related injury, or falls. The results of this study are novel; to our knowledge, no other randomized trial of compliant flooring has been conducted in LTC. Three previous nonrandomized studies of compliant flooring were conducted in LTC [14,15,17], and two studies were conducted in acute care [16,24]. In contrast to the results of the current trial, previous studies suggested a protective effect of complaint flooring against fall-related injuries. However, previous studies involved considerably smaller sample sizes, shorter follow-up durations, fewer events, less rigorous study designs, other types of compliant flooring, and less control of confounding factors.

We hypothesized compliant flooring would reduce the incidence of fall-related injuries by lowering impact forces experienced by the body below injury thresholds. Given the results of this study, however, it appears that fall events contained enough mechanical energy that differences in stiffness between compliant and control floors were not great enough to prevent the occurrence of injuries. Rather, risk for fall-related injury appeared to be governed by factors other than the flooring we investigated. We attempted to minimize differences in physiological risk factors for falls and injuries by randomly allocating residents to compliant or control flooring, and indeed the characteristics of residents, in terms of physical and cognitive status, disease diagnoses, medications, and fall and fracture history, were similar among residents living in compliant flooring and control rooms. We also found through our audits that room characteristics (e.g., area, presence of grab bars) were similar between compliant flooring and control rooms. However, we could not control for the mechanics of falls or the exact location of falls within resident rooms. While beyond the scope of our study, further analysis may reveal whether clinical characteristics of residents were associated with risk or rate of fall-related injury.

The influence of compliant flooring on fall-related injuries may have been diminished by more dominant factors related to characteristics of the fall (such as height, direction, landing configuration, and nearby and held objects) [25,26], and characteristics of the faller (such as intactness of fall protective responses and resistance of tissues to injury) that affected risk for impact and injury to vulnerable locations such as the head, which was the most common site for serious injury in both groups. Since almost all falls were unwitnessed, it was not possible to know which body parts experienced impact during falls and whether there was impact with walls, furniture, or other objects during fall descent that would render floor stiffness less important in determining risk for injury. Given the advanced age, end-of-life status, and physical and cognitive vulnerability of residents in LTC, variability in fall severity may be especially high, and the ability of flooring to modify fall-related injury risk may be inherently low in this setting.

Our findings address stakeholder calls for knowledge about the influence of compliant flooring on balance and falls [18] by showing that the compliant flooring we examined did not affect risk for falls. Previously, a 1-year cluster randomized controlled trial in geriatric wards at eight hospitals suggested a small increase in rate of falls and risk of falling on compliant relative to control flooring [24]. A 31-month prospective observational study in a subacute older persons’ hospital health ward tested three types of compliant flooring and reported no difference in rate of falls between compliant and control flooring [16]. Results from our study of 1907 falls provide strong evidence that the compliant flooring we tested does not influence falls in the LTC setting.

Future research on fall-related injury prevention in LTC is needed to reduce morbidity from falls. Given the prominence of serious head injuries observed in the current study, one future direction is to design and evaluate wearable protective head gear that addresses the needs and preferences of residents as well as their families and care givers. Another direction is to investigate the feasibility and effectiveness of novel technologies with superior impact force attenuation properties than the flooring we tested, such as impact-mitigating thermoplastics used in the automobile and defense industries. Such technologies may be useful as coverings for walls, edges of furniture, and ground surfaces in resident rooms and other areas of LTC sites. To be acceptable in LTC, such technologies must reduce risk for resident injury without increasing risk for resident falls or ergonomic issues for staff, and their installation must be feasible in areas with moisture, including bathrooms.

This study has several strengths. To our knowledge, it is the largest and most methodologically rigorous randomized trial of compliant flooring undertaken to date. The brand of compliant flooring that was evaluated (SmartCells) provides substantial impact force attenuation during biomechanical testing in laboratory experiments and was the only brand of compliant flooring that had been tested extensively for effects on balance and mobility at the time of trial onset. Finally, this study occurred in an active LTC home, and there were no efforts to interfere with regular practice; thus, the results estimate how compliant flooring performs under real-world conditions.

This study has certain limitations. First, the results of this trial are specific to 2.54 cm SmartCells compliant flooring installed in LTC resident rooms and may not generalize to other brands or models of compliant flooring or to installations in other areas of LTC sites such as hallways or common areas. Second, the LTC site continued to provide usual fall and injury prevention interventions throughout the study, so competing interventions may have been added, removed, or modified in specific rooms or for specific residents during the study, which may have altered the effect of compliant flooring. To this end, hip protector use was high among study residents. The most recent Cochrane review reported that hip protectors lead to a small reduction in risk of hip fracture in LTC, though they have no beneficial effect on other fractures and may cause a small increase in risk of pelvic fracture [27]. Low compliance with hip protectors in LTC is a barrier to their effectiveness for hip fracture prevention. Some evidence shows that specific types of hip protectors substantially reduce the risk of hip fracture when worn at the time of a fall [28–31]. Therefore, the results of the current trial may not generalize to LTC sites with different rates of hip protector compliance. Third, the rigid plywood control floor may have provided a small amount of impact force attenuation relative to concrete; thus, our results may have been different if concrete was the comparator. Fourth, falls on compliant flooring may have led to lower levels of pain than falls on control flooring, but we did not assess pain severity after falls. Fifth, some emergency department visits were for suspected injuries that were not confirmed after hospital investigation but were still classified as serious pain injuries. Finally, we found 21.2% of serious injuries were to the head. However, consistent with our previous studies of falls in LTC involving head impact, none of these were classified as concussions or brain injuries [26]. We suspect this reflects underreporting of concussions due to falls in LTC, in which 54.9% of residents in our study had dementia diagnoses. Cognitively impaired residents may be less likely to report hitting their head in a fall or communicate symptoms, and improved approaches are required for distinguishing the cognitive effects of head impact from baseline dementia. These challenges with detection may have prevented us from measuring the true effect of compliant flooring on fall-related concussions.

In conclusion, our study suggests that the type of compliant flooring we tested is not effective for preventing serious fall-related injuries in LTC. The results of this study will inform policies, programs, and practices for fall injury prevention in LTC that seek to improve resident quality of life, healthcare, and wellbeing.

## Supporting information

S1 Table Comparison of serious fall-related injuries between compliant flooring INT and control flooring CON groups in the FLIP Study, 2013–2017. Residents with fall rates in the top fifth percentile excluded. CON, Control; FLIP, Flooring for Injury Prevention; INT, intervention.
(DOCX)

S2 Table Subgroups analyses. Cells contain *p*-values for interaction terms.
(DOCX)

S3 Table Comparison of secondary outcomes between compliant flooring INT and control flooring CON groups in the FLIP Study, 2013–2017. Residents with fall rates in the top fifth percentile excluded. CON, control; FLIP, Flooring for Injury Prevention; INT, intervention.
(DOCX)

S1 Protocol Study protocol for the FLIP Study. FLIP, Flooring for Injury Prevention.
(PDF)

S1 Checklist CONSORT checklist for the FLIP Study. FLIP, Flooring for Injury Prevention.
(PDF)

## Abbreviations

ADL: activities of daily living
CADTH: Canadian Agency for Drugs and Technologies in Health
CVD: Cardiovascular disease
FLIP: Flooring for Injury Prevention
HR: hazard ratio
LTC: long-term care
MDS: Minimum Data Set
OR: odds ratio
RR: rate ratio
TIA: transient ischemic attack
