# Supplementary material for: The Flooring for Injury Prevention (FLIP) Study of compliant flooring for the prevention of fall-related injuries in long-term care: A randomized trial
Source: PLoS Med. 2019 Jun 24;16(6):e1002843. doi: 10.1371/journal.pmed.1002843 (PMC6590787; doi:10.1371/journal.pmed.1002843)
Supplement: S1 Table — Residents with fall rates in the top fifth percentile excluded. CON, Control; FLIP, Flooring for Injury Prevention; INT, intervention. (DOCX) [file pmed.1002843.s001.docx]

**S1 Table. Comparison of serious fall-related injuries between compliant flooring INT and control flooring CON groups in the FLIP Study, 2013–2017.** Residents with fall rates in the top fifth percentile excluded. CON, Control; FLIP, Flooring for Injury Prevention; INT, intervention

|  | **Compliant Flooring INT (n=169)** | | **Control Flooring CON (n=167)** | | **Base Model^a^** | | **Multivariable Model^b^** | |
| --- | --- | --- | --- | --- | --- | --- | --- | --- |
| **Endpoint** | **Events** | **Risk** | **Events** | **Risk** | **OR (95% CI)** | ***p*** | **OR (95% CI)** | ***p*** |
| ≥1 serious fall-related injury | 20 | 11.8 | 22 | 13.2 | 0.92 (0.48,1.78) | 0.814 | 0.97 (0.49,1.94) | 0.940 |
| ≥2 serious fall-related injuries | 8 | 4.7 | 12 | 7.2 | 0.67 (0.25,1.68) | 0.402 | 0.75 (0.27,1.98) | 0.558 |
|  |  |  |  |  |  |  |  |  |
|  | **Events** | **Rate** | **Events** | **Rate** | **RR (95% CI)** | ***p*** | **RR (95% CI)** | ***p*** |
| Number of serious fall-related injuries/1,000 bed nights | 33 | 0.327 | 44 | 0.406 | 1.07 (0.46, 2.53) | 0.857 | 1.22 (0.53, 2.81) | 0.619 |
| Number of serious fall-related injuries/fall | 33 | 0.047 | 44 | 0.053 | 0.98 (0.45, 2.10) | 0.950 | 1.09 (0.50, 2.35) | 0.819 |
| Number of falls with ≥1 serious fall-related injury/1,000 bed nights | 24 | 0.238 | 31 | 0.286 | 0.88 (0.46, 1.64) | 0.680 | 0.94 (0.50, 1.76) | 0.854 |
|  |  |  |  |  |  |  |  |  |
|  | **Events** | **Rate** | **Events** | **Rate** | **HR (95% CI)** | ***p*** | **HR (95% CI)** | ***p*** |
| Time to first serious fall-related injury^c^ | 20 | 0.214 | 23 | 0.248 | 0.87 (0.48, 1.58) | 0.640 | 0.88 (0.47, 1.64) | 0.671 |

^a^ Includes main effect term for intervention group (1=INT, 0=CON). For binary logistic models that generated ORs, bed nights of follow-up was a covariate. For negative binomial models that generated RRs, offset was specified as bed nights of follow-up for endpoint of number of serious fall-related injuries/1,000 bed nights, and offset was specified as number of falls for endpoint of number of serious fall-related injuries/fall.

^b^ Base model plus adjustment for baseline values for the following five covariates: age (<85, 85+ years), dementia, ≥1 fall in the past 180 days, antianxiety medication, and analgesic medication.

^c^ Rate expressed as events per 1,000 bed nights.

Abbreviations: CON, control; FLIP, Flooring for Injury Prevention, HR, hazard ratio; INT, intervention; OR, odds ratio; RR, rate ratio.
