# Supplementary material for: The Flooring for Injury Prevention (FLIP) Study of compliant flooring for the prevention of fall-related injuries in long-term care: A randomized trial
Source: PLoS Med. 2019 Jun 24;16(6):e1002843. doi: 10.1371/journal.pmed.1002843 (PMC6590787; doi:10.1371/journal.pmed.1002843)
Supplement: S2 Table — Cells contain p-values for interaction terms. (DOCX) [file pmed.1002843.s002.docx]

**S2 Table.** **Subgroups analyses.** Cells contain *p*-values for interaction terms.

|  | **Subgroup** | | | | | |
| --- | --- | --- | --- | --- | --- | --- |
| **Endpoint** | **Age  (<85, ≥ 85 yrs)** | **Sex (male, female)** | **Body mass index (<25, ≥ 25 kg/m^2)^** | **Dementia  (yes, no)** | **≥ 1 falls past  180 days  (yes, no)** | **Able to stand independently (yes, no)** |
| ≥1 serious fall-related injury | 0.676 | 0.492 | 0.552 | 0.669 | 0.162 | 0.627 |
| ≥2 serious fall-related injuries | 0.830 | 0.270 | 0.749 | 0.092 | 0.537 | 0.988 |
| Number of serious fall-related injuries/1,000 bed nights | 0.265 | 0.100 | 0.649 | 0.178 | 0.689 | 0.156 |
| Number of serious fall-related injuries/fall | 0.118 | 0.179 | 0.520 | 0.541 | 0.546 | 0.515 |
| Number of falls with ≥1 serious fall-related injury/1,000 bed nights | 0.595 | 0.214 | 0.960 | 0.149 | 0.267 | 0.404 |
| Time to first serious fall-related injury | 0.518 | 0.611 | 0.497 | 0.498 | 0.110 | 0.585 |
