# Supplementary material for: The Flooring for Injury Prevention (FLIP) Study of compliant flooring for the prevention of fall-related injuries in long-term care: A randomized trial
Source: PLoS Med. 2019 Jun 24;16(6):e1002843. doi: 10.1371/journal.pmed.1002843 (PMC6590787; doi:10.1371/journal.pmed.1002843)
Supplement: S3 Table — Residents with fall rates in the top fifth percentile excluded. CON, control; FLIP, Flooring for Injury Prevention; INT, intervention. (DOCX) [file pmed.1002843.s003.docx]

**S3 Table.** **Comparison of secondary outcomes between compliant flooring INT and control flooring CON groups in the FLIP Study, 2013-2017.** Residents with fall rates in the top fifth percentile excluded. CON, control; FLIP, Flooring for Injury Prevention; INT, intervention

|  | **Compliant Flooring INT (n=169)** | | **Control Flooring CON (n=167)** | | **Base Model^a^** | | **Multivariable Model^b^** | |
| --- | --- | --- | --- | --- | --- | --- | --- | --- |
| **Minor Fall-Related Injury** | **Events** | **Risk** | **Events** | **Risk** | **OR (95% CI)** | ***p*** | **OR (95% CI)** | ***p*** |
| ≥1 minor fall-related injury | 75 | 44.4 | 74 | 44.3 | 1.08 (0.68, 1.71) | 0.740 | 1.26 (0.78, 2.04) | 0.356 |
| ≥2 minor fall-related injuries | 52 | 30.8 | 54 | 32.3 | 1.00 (0.61, 1.63) | 0.990 | 1.18 (0.70, 1.99) | 0.554 |
|  | **Events** | **Rate** | **Events** | **Rate** | **RR (95% CI)** | ***p*** | **RR (95% CI)** | ***p*** |
| Number of minor fall-related injuries/1,000 bed nights | 274 | 2.712 | 335 | 3.092 | 0.90 (0.61, 1·32) | 0.580 | 1.10 (0.75, 1.62) | 0.627 |
| Number of minor fall-related injuries/fall | 274 | 0.389 | 335 | 0.407 | 1.08 (0.83, 1·41) | 0.570 | 1.27 (0.97, 1.68) | 0.086 |
| Number of falls with ≥1 minor fall-related injury/1,000 bed nights | 180 | 1.782 | 253 | 2.335 | 0.78 (0.55, 1·12) | 0.170 | 0.93 (0.66, 1.32) | 0.656 |
|  | **Events** | **Rate** | **Events** | **Rate** | **HR (95% CI)** | ***p*** | **HR (95% CI)** | ***p*** |
| Time to first minor fall-related injury^c^ | 75 | 1.16 | 75 | 1.18 | 0.98 (0.71, 1·35) | 0.890 | 1.03 (0.74, 1.43) | 0.874 |
|  |  |  |  |  |  |  |  |  |
| **Any Fall-Related Injury** | **Events** | **Risk** | **Events** | **Risk** | **OR (95% CI)** | ***p*** | **OR (95% CI)** | ***p*** |
| ≥1 fall-related injury | 79 | 46.7 | 80 | 47.9 | 1.02 (0.65, 1.60) | 0.940 | 1.15 (0.71, 1.85) | 0.574 |
| ≥2 fall-related injuries | 55 | 32.5 | 58 | 34.7 | 0.97 (0.60, 1.58) | 0.910 | 1.13 (0.68, 1.91) | 0.632 |
|  | **Events** | **Rate** | **Events** | **Rate** | **RR (95% CI)** | ***p*** | **RR (95% CI)** | ***p*** |
| Number of fall-related injuries/1,000 bed nights | 307 | 3.039 | 379 | 3.499 | 0.94 (0.64, 1.38) | 0.740 | 1.13 (0.78, 1.66) | 0.515 |
| Number of fall-related injuries/fall | 307 | 0.431 | 379 | 0.457 | 1.14 (0.86, 1.50) | 0.350 | 1.25 (0.96, 1.64) | 0.103 |
| Number of falls with ≥1 fall-related injury/1,000 bed nights | 193 | 1.910 | 272 | 2.511 | 0.81 (0.57, 1.15) | 0.220 | 0.93 (0.67, 1.31) | 0.677 |
|  | **Events** | **Rate** | **Events** | **Rate** | **HR (95% CI)** | ***p*** | **HR (95% CI)** | ***p*** |
| time to first fall-related injury^c^ | 79 | 1.23 | 81 | 1.35 | 0.92 (0.68, 1.26) | 0.610 | 0.93 (0.68, 1.29) | 0.659 |
|  |  |  |  |  |  |  |  |  |
| **Falls** | **Events** | **Risk** | **Events** | **Risk** | **OR (95% CI)** | ***p*** | **OR (95% CI)** | ***p*** |
| ≥1 fall | 113 | 66.9 | 111 | 66.5 | 1.10 (0.68, 1.78) | 0.700 | 1.21 (0.73, 2.00) | 0.464 |
| ≥2 falls | 79 | 46.7 | 88 | 52.7 | 0.83 (0.52, 1.30) | 0.411 | 0.86 (0.54, 1.39) | 0.543 |
|  | **Events** | **Rate** | **Events** | **Rate** | **RR (95% CI)** | ***p*** | **RR (95% CI)** | ***p*** |
| Number of falls/1,000 bed nights | 703 | 6.958 | 823 | 7.597 | 0.91 (0.67, 1.23) | 0.520 | 0.95 (0.69, 1.30) | 0.743 |
|  | **Events** | **Rate** | **Events** | **Rate** | **HR (95% CI)** | ***p*** | **HR (95% CI)** | ***p*** |
| Time to first fall^c^ | 112 | 2.38 | 110 | 2.39 | 0.99 (0.76, 1.28) | 0.920 | 0.94 (0.72, 1.24) | 0.669 |

^a^ Includes main effect term for intervention group (1=INT, 0=CON). For binary logistic models that generated ORs, bed nights of follow-up was a covariate. For negative binomial models that generated RRs, offset was specified as bed nights of follow-up for endpoint of number of serious fall-related injuries/1,000 bed nights, and offset was specified as number of falls for endpoint of number of serious fall-related injuries/fall.

^b^Base model plus adjustment for baseline values for the following five covariates: age (<85, 85+ years), dementia, ≥1 fall in the past 180 days, antianxiety medication, and analgesic medication.

^c^Rate expressed as events per 1,000 bed nights.

Abbreviations: CON, control; FLIP, Flooring for Injury Prevention; HR, hazard ratio; INT, intervention; OR, odds ratio; RR, rate ratio.
