# Supplementary material for: The Flooring for Injury Prevention (FLIP) Study of compliant flooring for the prevention of fall-related injuries in long-term care: A randomized trial
Source: PLoS Med. 2019 Jun 24;16(6):e1002843. doi: 10.1371/journal.pmed.1002843 (PMC6590787; doi:10.1371/journal.pmed.1002843)
Supplement: S1 Protocol — FLIP, Flooring for Injury Prevention. (PDF) [file pmed.1002843.s004.pdf]

Study Protocol for the Flooring for Injury Prevention (FLIP) Study:

A Randomized Controlled Trial in Long-Term Care

Chantelle C. Lachance[1,2], Fabio Feldman[1,2,3], Andrew C. Laing[4], Pet Ming Leung[3], Stephen N. Robinovitch[1,2], Dawn C. Mackey[1,2]

1. Department of Biomedical Physiology and Kinesiology, Simon Fraser University, Burnaby, Canada
2. Centre for Hip Health and Mobility, University of British Columbia, Vancouver, Canada
3. Fraser Health Authority, Surrey, Canada
4. Department of Kinesiology, University of Waterloo, Waterloo, Canada

Corresponding Author:

Dawn C. Mackey, PhD

Aging and Population Health Lab,

Department of Biomedical Physiology and Kinesiology

K9625 Shrum Science Centre

Simon Fraser University

8888 University Drive, Burnaby, British Columbia, Canada V5A 1S6

Phone: 778.782.9330 | Fax: 778.782.3040 | Email: dmackey@sfu.ca

KEYWORDS: low stiffness flooring, older adults, residential care, fall injuries, hip fracture

WORD COUNT (excluding title page, abstract, references, figures, tables): 5432 words

ABSTRACT

Background. A promising strategy for reducing the incidence and severity of fall-related injuries in long-term care (LTC) is to decrease the ground surface stiffness, and the subsequent forces applied to the body parts at impact, through installation of compliant flooring that does not substantially affect balance or mobility. Definitive evidence of the effects of compliant flooring on fall-related injuries in LTC is lacking. The Flooring for Injury Prevention (FLIP) Study is designed to address this gap.

Methods. The FLIP Study is a 4-year, parallel-group, 2-arm, randomized controlled superiority trial of flooring in 150 resident rooms at a LTC site. The primary objective is to determine whether compliant flooring reduces serious fall-related injuries relative to control flooring. Intervention (2.54cm SmartCells compliant; 74 rooms) and control (2.54cm plywood; 76 rooms) flooring were installed over top of existing concrete floors and covered with identical 2.00mm vinyl. The primary outcome is serious fall-related injury, defined as any impact-related injury due to a fall in a study room that results in Emergency Department visit or hospital admission. Secondary outcomes include minor fall-related injury, any fall-related injury, falls, number of fallers, fractures, and health care utilization and costs for serious fall-related injuries. Randomization of study rooms, and residents in rooms, was stratified by residential unit, and flooring assignments were concealed. Outcome ascertainment began September 2013.

Discussion. Results from the FLIP Study will provide evidence about the effects of compliant flooring on fall-related injuries in LTC and will guide development of safer environments for vulnerable older adults.

Trial registration. Clinicaltrials.gov identifier number NCT01618786

BACKGROUND

Falls and the injuries they cause are a major public health concern for older adults. In this population, falls are the leading cause of injury-related death and unintentional injury, including over 90% of hip[1 2] and wrist fractures[3] and 80% of traumatic brain (“head”) injuries.[4 5] Approximately 20% of hip fracture patients die within one year of fracture and 50% do not return to their pre-fracture level of mobility, quality of life, or independence.[6-8] Traumatic brain injuries have tripled in incidence over the past decade,[4 9 10] and they cause over half of all fall-related deaths in older adults.[11] Non-fatal traumatic brain injuries commonly result in long-term cognitive, emotional, and functional impairments.[12] In Canada, falls and the injuries they cause in older adults cost over $3 billion annually.[13]

The long-term care (LTC) setting is a particularly high-risk environment for falls. LTC refers to sites for older adults where personal and nursing care is provided on a 24-hour basis (e.g. nursing homes, residential care facilities).[14] Approximately 60% of LTC residents fall at least once per year, and 30% of falls in LTC result in injury, rates that are two to three times higher than among older adults living independently.[15-17] Moreover, hip fractures from falls are 10 times more likely among LTC residents than community-dwelling older adults,[15 17 18] and approximately 25% of traumatic brain injuries from falls in older adults occur in LTC.[4]

Preventing falls among older adults in LTC remains a significant challenge,[19-21] in part because LTC residents are more frail, have more chronic disease, and are more likely to have significant cognitive and physical impairments than community-dwelling older adults. The most recent Cochrane Review showed that Vitamin D

supplementation leads to modest reductions in fall rates in LTC,[19] but concluded there is a lack of definitive evidence for the effectiveness of other fall prevention strategies in LTC, either single or multi-factorial.[19] A more recent systematic review and meta-analysis found that multi-factorial fall prevention programs in LTC reduced the number of falls by 33% and recurrent fallers by 21%.[20] However, because multi-factorial fall prevention programs are typically delivered by multi-disciplinary teams and customized to individual risk factors, implementation tends to be challenging, time-consuming, and costly.[20]

Efforts to prevent fall-related injuries in LTC have focused largely on fracture prevention. Recent Canadian recommendations for fracture prevention in LTC include Vitamin D and calcium supplementation, use of hip protectors, exercise, multifactorial interventions to prevent falls, and pharmacologic therapies.[22] Given the serious consequences of hip fracture among residents, hip fracture prevention has been an area of focus in LTC, and studies have demonstrated that hip protectors prevent hip fracture when they are worn, but poor adherence is a major barrier to their effectiveness.[23] In light of the above evidence, and given the high probability for falls to result in injury in LTC,[18] it is essential to develop new approaches for preventing fall-related *injuries* in LTC to complement existing strategies for preventing falls.

A promising strategy for reducing the incidence and severity of fall-related injuries in LTC is to decrease the stiffness of the ground surface, and the subsequent forces applied to body parts at impact, through the implementation of compliant flooring systems.[24-27] Laboratory studies have demonstrated that compliant flooring (also called ‘low stiffness flooring’, ‘energy absorbing flooring’, ‘safety flooring’, shock

absorbing flooring, ‘impact absorbing flooring’, and ‘low impact flooring’) can reduce the peak force applied to the hip during a simulated sideways fall by up to 35%[24] and to the head during a simulated backwards fall by up to 60%,[28] without substantially impairing balance or mobility of older adults.[24 29-32] Laboratory studies have also demonstrated that compliant flooring is particularly effective at reducing impact forces to the hip among adults with low body mass index, such as frail older adults in LTC.[33-35] Compliant flooring is a passive intervention approach that (once installed) does not rely upon user adherence, and unlike hip protectors, compliant flooring has the potential to reduce the frequency of all serious fall-related injuries, including hip and wrist fractures and traumatic brain injuries. Indeed, compliant flooring represents one of the few options that may be feasible and effective for preventing fall-related traumatic brain injuries. Commercially available compliant flooring is optimized for use in health care settings by meeting strict hygiene and durability criteria. Nevertheless, few LTC sites have implemented compliant flooring systems specifically designed to reduce the severity of fall impacts, in part because sufficient clinical evidence that compliant flooring reduces fall-related injuries has not been available.

Preliminary clinical findings suggest that compliant flooring may reduce fall-related injuries in LTC. A 2.5-year retrospective study at a U.S. LTC site found there was a non-significant trend for fewer bruises and abrasions from falls on compliant flooring (2.54 cm SmartCells installed in two resident bedrooms and bathrooms) than falls on standard flooring.[36] While two falls on standard flooring resulted in fracture, no falls on compliant flooring resulted in fracture.[36] Further, a non-randomized study of female residents at a Swedish LTC site installed compliant flooring (1.25 cm Kradal) in

350 m^2 of a single ward, including some resident rooms and common areas but no bathrooms.[37] During 2.5 years of prospective falls surveillance, 16.9% of falls (13 of 77) on compliant flooring were injurious compared to 30.3% of falls (77 of 254) on control flooring (vinyl, linoleum, and ceramic tile all with concrete underlay), which resulted in a significant 59% reduction (95% CI: 20-80%) in risk for fall-related injury after adjustment for individual-level covariates. However, 80% of fall-related injuries were of minor severity (e.g., distinct pain, bruising, swelling), and there was insufficient statistical power to test the effect of compliant flooring on more serious fall-related injuries.

Preliminary findings also suggest that compliant flooring may reduce fall-related injuries in hospital settings. In an unblinded, pilot cluster randomized controlled trial in the U.K., geriatric wards at eight hospitals were allocated to compliant (8.3 mm vinyl Tarkett Omnisports EXCEL) or control (standard) flooring.[38] During one year of outcome monitoring, 23% of falls (8 of 35) were injurious in intervention wards compared to 42% of falls (14 of 33) in control wards, which resulted in a non-significant 42% reduction (95% CI: 81% reduction – 91% increase) in the incidence of fall-related injuries. No moderate or major severity injuries occurred in intervention wards, while six occurred in control wards. In a non-randomized study at a New Zealand hospital, three types of compliant flooring were installed in a total of six bedrooms (with a total of 12 beds) on a single ward.[39] Over 6 months, 40.4% of falls (21 of 52) on standard flooring were injurious compared to 23.8% (5 of 21) on compliant flooring.

The above evidence suggests that compliant flooring may be an effective intervention for preventing fall-related injuries in LTC and hospital settings, but the

number of falls and fall-related injuries observed within individual studies have been small, precluding definitive conclusions. In particular, no randomized controlled trial has been sufficiently powered to test the effectiveness of compliant flooring for reducing fall-related injuries in LTC. Such evidence is needed to inform fall injury prevention strategies in LTC and to aid in the design of new and safer sites. The Flooring for Injury Prevention (FLIP) Study is a 4-year randomized controlled trial to determine whether compliant flooring reduces fall-related injuries among older adults living in the LTC setting.

The primary objective of the FLIP Study is to determine whether compliant flooring reduces serious fall-related injuries in LTC residents relative to control flooring. The secondary objectives of the FILP Study are to determine whether compliant flooring reduces minor fall-related injuries, all fall-related injuries (i.e., minor and serious), fractures, and health care utilization and costs due to serious fall-related injuries, and whether compliant flooring increases number of falls and fallers among LTC residents relative to control flooring.

METHODS

Design and setting

The FLIP Study is a 4-year, parallel-group, 2-arm, randomized controlled superiority trial of flooring in 150 single-occupancy resident rooms at a single LTC site (comprised of 236 single-occupancy resident rooms) in British Columbia, Canada (Figure 1).

Eligibility

We focused on modifying flooring in resident rooms as opposed to common areas (e.g., dining halls, hallways), as the majority of falls at LTC sites (~75%) occur in resident rooms.[40] Installation of intervention and control flooring in resident rooms and adjacent hallways resulted in floors being raised by 2.54 cm; to accommodate these changes, four-foot long transition ramps were installed between the raised hallways and non-renovated common areas. Thus, resident rooms where the floor could not feasibly be raised by 2.54 cm (n = 37) were not eligible for inclusion. This often occurred at the ends of hallways there was insufficient space to install transition ramps. In addition, resident rooms on the third floor of the LTC site (n = 49) were excluded as most residents on this floor were non-ambulatory (wheelchair bound). This left 150 resident rooms for inclusion.

Randomization procedure

Randomization of study rooms, and therefore residents living within those rooms, was performed before flooring installation began and was stratified by residential unit (three units on the first floor and one unit on the second floor) in blocks of four resident rooms with 1:1 allocation. Construction approaches dictated that a maximum of two control and two intervention resident rooms could be renovated per day; blocks of four rooms ensured this balance. The randomization sequence was computer generated and shared only with the flooring installation team and an on-site project manager at the LTC site who was not a member of the research team responsible for data collection and analysis. Flooring assignments were concealed from LTC residents and their

families, LTC staff, and members of the FLIP Study research team who were involved in data collection and analysis. In addition, the flooring installation team, LTC residents and their families, and LTC staff remained unaware of the study's specific research questions.

Study intervention

Intervention flooring (compliant flooring)

Intervention flooring (2.54 cm SmartCells, SATECH Inc., Chehalis, WA, USA) was installed in 74 resident rooms (including living, bathroom, and closet areas) over top of existing concrete floors (total area of SmartCells installed = 1151.9 m^2; average area of SmartCells installed per resident room = 15.6 m^2). SmartCells is a synthetic rubber floor system (50 durometer surface hardness; density = 1120 kg/m^3) comprised of a continuous surface layer supported by an array of cylindrical rubber columns, 0.14 cm in diameter and spaced at 0.19 cm intervals apart.[29]

Control flooring

Control flooring (2.54 cm thick plywood, considered a rigid floor) was installed in 76 resident rooms as well as hallways adjacent to resident rooms, over top of existing concrete floors. Plywood provides only a small degree of force attenuation (1.6%) during impact from simulated falls relative to concrete.[26 27] As there is no established effective flooring intervention for fall-injury prevention in the LTC setting, plywood was regarded as a placebo control.

Vinyl overlay

Intervention and control flooring were covered with identical hospital-grade vinyl (2.0 mm AltroSmooth Ethos, Altro, Mississauga, ON, Canada). To minimize the likelihood of LTC staff (part of the outcome assessment team) being influenced in their reporting of fall-related injuries (e.g., location, type, severity) by any perceived or real knowledge about the flooring on which falls occurred, details about the types and numbers of floors being evaluated were not communicated to LTC staff, residents, or families.

Site renovations

The FLIP Study research team had several planning meetings with the LTC site administrators prior to the renovations. The hired contractor had previously completed successful large-scale renovation projects at the LTC site and was skilled at working within an active LTC site. The LTC administrators and contractor jointly developed a detailed 12-week renovation plan (April 1 to June 21, 2013) designed to minimize disruption to residents and LTC staff. Each week, the contractor installed temporary partitions in the hallway around the block of rooms under renovation to conceal the flooring assignments from LTC staff and residents. During the week that resident rooms in a given hallway were under renovation, all resident rooms were vacated from early morning until evening, and residents moved to common areas such as dining halls and lounges for the day where semi-private space was available to accommodate napping. Throughout the renovation phase, the LTC site provided additional daytime activity

programming, and resident families were informed well in advance of the renovation schedule so they could plan off-site trips.

Study outcomes

Primary outcome

The primary outcome for the FLIP Study is serious fall-related injury, defined as any impact-related injury due to a fall in a study room that results in an Emergency Department visit or a hospital admission. Serious fall-related injuries are classified as fractures or soft tissue injuries. Fractures are classified based on location (e.g. hip, wrist). Soft tissue injuries are classified by type as hematoma, dislocation, laceration/cut, sprain/strain, contusion/bruise, swelling, pain, abrasion, or other. To avoid inclusion of less serious injuries that might not receive external medical care at other LTC sites (an issue of external validity), we require the following criteria be met for fall-related injuries that lead to an Emergency Department visit or hospital admission: (i) that fractures are confirmed via X-ray; (ii) that lacerations are sutured; and (iii) a procedure indicating treatment or a diagnostic evaluation for hematomas, sprains/strains, contusions/bruises, swelling, pain, abrasions, or other.

When a resident is transferred to a hospital following a fall for a reason other than an impact-related injury (e.g., confusion, agitation, low blood glucose, low hemoglobin, possible urinary tract infection), this is not considered to be a fall-related injury.

Secondary outcomes

Secondary outcomes for the FLIP Study include: (i) minor fall-related injury,

defined as any impact-related injury due to a fall in a study room that does not result in an Emergency Department visit or a hospital admission; (ii) any fall-related injury (i.e., minor or serious); (iii) falls in study rooms; (iv) number of fallers in study rooms; (v) fractures in study rooms; and (vi) health care utilization and costs due to serious fall-related injuries in study rooms. Health care utilization will be determined by accounting for health care resources used: hospitalizations (transfers, emergency room visits, admissions, length of stay); health professional time stratified by provider (e.g., orthopedic surgeon, physical therapist, general practitioner); radiologic evaluations (e.g., CT, X-ray, ultrasound), laboratory evaluations, and other diagnostic evaluations.

Measurement of study outcomes

Study outcomes will be monitored for 4 years (September 2013 – August 2017) (Figure 2). Each resident fall that occurs at the LTC site is reported by nursing staff on an incident form within 24 hours of the time of the fall. A fall is defined as an unexpected event in which a resident comes to rest on the ground, floor, or lower level [41]. From incident forms, trained research assistants ascertain the date, time, location, cause, and circumstances of each fall, as well as details about the nature and extent of any resulting injuries apparent at the time of the fall. From incident forms we also ascertain Emergency Department visits and/or hospital admissions that are due to fall-related injuries. From resident charts, we ascertain additional details about fall-related injuries that become available up to 7 days after a fall incident. Information from incident forms, resident charts, and hospital records (if applicable) are used to ascertain the nature and extent of fall-related injuries, including injury type, location, and severity (Figure 2).

For the planned cost-effectiveness analyses, health care utilization for serious fall-related injuries is extracted from LTC site records by trained research assistants and a clinical nurse educator with expertise in fall and injury prevention. Health care costs will be calculated by multiplying the frequency of each service by the prevailing local cost.

Other study measures

Resident characteristics

Several resident characteristics may increase or decrease their risk for falls and injuries. We will attempt to ascertain the following characteristics of residents living in study rooms at the beginning of the follow-up period: sex, age, body mass index, length of stay at the LTC site, fall and fracture history, pain history, mobility status (independent, walker, wheelchair), medical conditions, cognitive function, mood and behavior, visual function, dependence in activities of daily living, gait function (e.g., rated as unsteady), sitting and standing balance, number and types of medications, use of physical restraint (e.g., lap belt in wheelchair), and advanced directives to not resuscitate. We will ascertain these characteristics via retrospective review of residents' Minimum Data Set (MDS 2.0, interRAI Corporation 1999), which is an extensive observational assessment of medical, functional, psychological, and cognitive status[42 43] completed annually and updated quarterly for all residents in LTC sites in British Columbia as mandated by the provincial Ministry of Health. We will update resident characteristics annually throughout the study follow-up period, as they may change over time because of change in resident status and resident turnover.

Room characteristics

Following flooring installation in intervention and control rooms and prior to the start of outcome surveillance, we ascertained room characteristics that could influence risk for falls and injury via room audits. For each study room we assessed the square footage of the room, square footage of the bathroom, room layout, whether the bathroom was open or closed off to the room, number of falls and fall-related injuries in the past 12 months, and number of unique residents in the past 12 months. We also determined the presence and location of fall mats, presence of grab bars, presence and type of adjustable bedrails, type of bed, presence of a ceiling lift, presence and location of furniture, and presence of any hazards (e.g., cables on floor, furniture with protruding legs, clutter in walkway areas). Room characteristics will be updated each time a new resident moves into a study room (i.e., new admission or internal move).

Data management

Fall data from incident forms are entered directly into the study database by trained research assistants. Injury data are transcribed from incident forms, resident charts, and hospital records (if applicable) onto study data collection forms and then entered into study databases by trained research assistants. To ensure data quality, all data are doubly entered and checks are done regularly to identify and correct errors. Where possible, data entry spreadsheets contain lists of valid codes and corresponding descriptions of codes. All original data are kept on file at the LTC site. De-identified data sets are transmitted to and stored at the study coordinating centre.

Statistical considerations

The FLIP Study was powered for the primary outcome of serious fall-related injury. Decision makers within the local health authority indicated that a 35-40% reduction in the rate of serious fall-related injuries in intervention versus control rooms would be deemed clinically important. Based on 2011 and 2012 fall injury data from the LTC site, we projected a 1-year cumulative incidence of serious fall-related injury for the control arm of 16.6%, which equates to a 4-year cumulative incidence of 66.2%. Under these assumptions, the FLIP Study will have 88% power to detect a 40% reduction and 78% power to detect a 35% reduction in the rate of serious fall-related injuries between intervention and control arms with a two-sided alpha of 0.05. Non-compliance and loss to follow-up will not apply to this environmental intervention.

The primary outcome will be tested with an intent-to-treat approach using a two-tailed significance level of 0.05. Resident will be the unit of analysis. The mean number of serious injuries per fall will be compared between residents living in rooms with intervention versus control flooring using a Poisson generalized linear mixed model (GLMM) to estimate a risk ratio and corresponding 95% CI, with adjustment for (i) time-varying covariates (e.g., room and resident characteristics) that are hypothesized to be associated with risk for fall-related injury, (ii) residential village (our unit of stratification for randomization) to account for potential clustering effects, and (iii) random resident and room effects to account for potential residual confounding from unmeasured covariates. As a sensitivity analysis to verify that the data reasonably meet the assumptions of the Poisson GLMM, we will also fit generalized estimating equations

(which have fewer distributional assumptions than Poisson GLMM) to estimate risk ratios and corresponding 95% confidence intervals. Robust standard errors will be used to account for potential correlation from multiple falls per resident. We will also compare time to first serious fall-related injury between groups using a Cox proportional hazards regression model with the same adjustment as described above to estimate a hazard ratio and corresponding 95% CI.

Secondary injury outcomes will also be analyzed with an intent-to-treat approach using a two-tailed significance level of 0.05. Mean number of minor injuries per fall, mean number of all (minor + serious) injuries per fall, and mean number of fractures per fall will be compared between groups using a Poisson GLMM and generalized estimating equations with robust standard errors, as described for the primary outcome. Time to first event (minor fall-related injury, any fall-related injury, fall, fracture) will be compared between groups using separate Cox proportional hazards regression models. Probability of falling on a given day will be compared between groups using a binary GLMM or a recurrent event model (if necessary) to estimate a risk ratio and corresponding 95% CI. For each of these secondary outcomes, we will fit models adjusted for (i) time-varying covariates (e.g., room and resident characteristics) that are hypothesized to be associated with risk for fall-related injury, (ii) residential village (our unit of stratification for randomization) to account for potential clustering effects, and (iii) random resident and room effects to account for potential residual confounding from unmeasured covariates.

Data analysts and investigators will be blinded; they will finalize analysis plans, conduct analyses, and write the first draft of the manuscript before treatment codes are revealed.

The planned economic evaluation will assess the effectiveness of compliant flooring in reducing fall-related injuries in relation to the costs of the intervention. The main measure of effect will be the incremental cost-effectiveness ratio (ICER). We will calculate the incremental cost per fall-related injury prevented by compliant flooring compared with control flooring from a Canadian Health Care System perspective. All hospital admission-related costs will be based on a fully allocated cost model of a tertiary care hospital and the remainder from the British Columbia Medical Services Plan guide to fees. Uncertainty in the estimation of the costs and effectiveness will be modeled by nested imputation and bootstrapping. This method accounts for the uncertainty due to both the missing values and the finite study sample size. For each run of imputation and bootstrapping, we will calculate (for each resident) the total cost and number of fall-related injuries. These outcomes will then be averaged for residents within each study arm. The contribution of different cost components to total costs will also be evaluated. Expected value (mean) of the cost and effectiveness outcomes along with their confidence intervals, plots on the cost effectiveness plane and acceptability curves will be generated.

Ethical considerations

The protocol for this study was approved by the Research Ethics Boards at Simon Fraser University (2013s0525) and the Fraser Health Authority (FHREB 2012-

059). Since the intervention is environmental (installation of compliant flooring), and the data collection activities are retrospective in nature and involve only secondary and de-identified data, we are not seeking consent from individual residents at the LTC site.

Study management and monitoring

The FLIP Study is being conducted at a single LTC site located in Burnaby, British Columbia, Canada. This is a non-for-profit, government-funded LTC site that provides 24-hour-a-day surveillance, personal care, and limited clinical care for older adult residents. All administrative and coordinating functions occur at Simon Fraser University in Burnaby, British Columbia, Canada. An independent three-member Data and Safety Monitoring Board (DSMB) has been appointed, consisting of experts in biostatistics, clinical trial design, and LTC, to review the study's progress and monitor the safety and scientific integrity of the study. We perceive the risks of this study to LTC residents to be minimal. We do not anticipate that intervention flooring will cause any increase in adverse events relative to control flooring. Resident deaths will be monitored prospectively, but death is common among LTC residents and will not be considered an adverse event of flooring. Infections may increase among residents during the flooring installation phase. We will monitor the number and type of resident infections during the renovation period (using data routinely collected by the LTC site), and compare rates during the renovation period to comparison periods prior to and after the renovation. As compliant flooring may increase rolling resistance,[38 44] we will also monitor the number and type of work-related musculoskeletal injuries sustained by staff (using data routinely collected by the LTC site), and compare rates during the study period to

comparison periods before the study. We will address any unintended effects of the interventions in collaboration with the LTC site and DSMB.

Access to data

Study investigators will have full access to all study data sets.

Dissemination policy

Results will be reported by study investigators through publication in peer-reviewed journals and disseminated more broadly via stakeholder partnerships.

DISCUSSION

The FLIP Study will evaluate the effect of compliant flooring on fall-related injuries among older adults in LTC. When completed, the FLIP Study will be the largest and longest randomized controlled trial of compliant flooring for fall-related injury prevention. Results of the FLIP Study are likely to have significant impact on the health and wellbeing of older adults. Specifically, the study will generate new knowledge about the efficacy and cost-effectiveness of compliant flooring for the prevention of fall-related injuries in LTC. Results should guide development of improved policies and programs for fall injury prevention, especially in LTC. Results will also inform planners and architects in the development of safer environments for vulnerable older adults, including hospitals, assisted living and LTC sites, community and recreation centres, and outdoor spaces (e.g., sidewalks, parks).

Our interdisciplinary research team, with expertise spanning epidemiology, biomechanics, biomedical engineering, health economics, and knowledge translation, is working alongside stakeholders in LTC and our local health authority whose collective knowledge and practical experience will contribute to the trial's success and to translating results into action.

As a potential intervention for fall-related injury prevention, compliant flooring is supported by over 10 years of laboratory studies that have demonstrated its biomechanical efficacy (i.e., impact force attenuation properties and minimal effect on balance and mobility) and support the development of clinical trials to test the effectiveness of compliant floors in high-risk environments, including LTC.[24 25 28 29] Recent preliminary studies suggest that compliant flooring may reduce fall-related injuries in LTC,[36 37] but these studies have used retrospective[36] and non-randomized[36 37] study designs, which leave open the opportunity for biased effect estimates, and they have been insufficiently powered to examine the effect of compliant flooring on the most serious and costly fall-related injuries. The FLIP Study will fill this gap in the evidence base.

Selection of the primary outcome

International guidelines published by the Prevention of Falls Network Europe (ProFaNE) recommend standardized methodology for conducting fall prevention trials, including standard methods of defining, collecting, and reporting falls.[41] In contrast, there are no standard methods of defining, collecting, or reporting fall-related injuries,[45] and a recent systematic review demonstrated that there is considerable

variability among randomized controlled trials in how fall-related injuries are defined, collected, and reported.[45] A main recommendation from their review was to define fall-related injuries using both symptoms and medical care use. Since medical care use is likely to vary across facilities and geographical locations (e.g., due to differences in risk aversion), using symptoms in addition to medical care use increases the generalizability (external validity) of the definition.

The FLIP Study definition of serious fall-related injury was designed to: (1) be consistent with the recommendation to define fall-related injury using both symptoms and medical care use; (2) match closely with the definition of fall-related injury used in a fall prevention cluster randomized controlled trial in LTC,[46] and (3) be similar to the 'Campbell definition' of fall-related injury,[47] a commonly used definition among existing fall prevention RCTs.[45] A potential limitation is that the FLIP Study definition of serious fall-related injury does not include traumatic brain injury, such as concussion, as a specific type of soft-tissue injury because these injuries are not typically noted on incident reports for falls involving head impact by LTC residents.[48] Given the high degree of baseline cognitive impairment among LTC residents, it is challenging to reliably ascertain traumatic brain injuries based on existing fall incident forms or resident charts at our partner LTC site,[49] and local hospitals rarely obtain computed tomography scans for fall-related hospital transfers from LTC. Further, because falls in the FLIP Study will not be captured on video, another limitation is that we may not be able to confirm whether fall-related injuries resulted from impact with an object other than the floor during descent (e.g., wall or furniture).

Selection of compliant flooring

Compliant flooring systems aim to maximize impact force attenuation while not being so soft that they impair balance and mobility. A number of commercially available compliant floors have been shown to substantially attenuate impact forces, including SmartCells, Forbo, Sorbashock, and Kradal.[24 28 33 35] We selected SmartCells for the FLIP Study for three main reasons: 1) it provided one of the highest degrees of impact force attenuation in our biomechanical tests; 2) to our knowledge, it is the only brand of compliant flooring that has been tested extensively for balance and mobility;[24 31 32] and 3) installation during retrofits is straightforward.

Competing interventions

Hip protector use is common at the LTC site and will not be altered for the purpose of the FLIP Study. This will likely result in an underestimation of the effect of compliant flooring on serious fall-related injuries (compared to LTC sites where hip protector use is lower), as evidence shows that hip protectors reduce hip fracture risk when they are worn at the time of a fall.[23] Hip protector use at the time of a fall is queried on incident forms, and we will ascertain this information when possible.

TRIAL STATUS

As of February 1, 2016 we have obtained ethical approval for the trial, registered the trial (Clinicaltrials.gov identifier: NCT01618786), and randomized study rooms. The LTC site completed installation of study flooring in June 2013. We have completed

baseline assessment of room characteristics and have monitored falls and fall-related injuries for 28 months (September 1, 2013 to December 31, 2015).

ACKNOWLEDGEMENTS

The authors acknowledge the partnership of New Vista Society Care Home for their support and facilitation of this study. The authors thank members of the Data Safety Monitoring Board (DSMB): Dr. Lynn Beattie, Ms. Gina Gaspard, and Dr. Carl Schwartz (chair). The authors thank Dr. Rachel Altman for assistance planning the statistical analysis and Dr. Carlo Marra for advising on the economic evaluation.

FUNDING

The study has received funding from the Canadian Institutes of Health Research (grant TIR-103945 to SNR); AGE-WELL, Inc., a Canadian National Centres for Excellence; Fraser Health Authority; Centre for Hip Health and Mobility; and New Vista Society Care Home. In addition, SATech provided SmartCells flooring, flooring installation materials (e.g., adhesive, tape, and transitions), and labour for flooring installation. CCL is supported by a Canadian Institutes of Health Research Frederick Banting and Charles Best Canada Graduate Scholarship and an AGE-WELL Graduate Student and Postdoctoral Award in Technology and Aging. DCM is supported by a Michael Smith Foundation for Health Research Scholar Award. The funding agencies had no direct role in the design and conduct of the study; the collection, management, analysis, or interpretation of the data; or the preparation, review, or approval of the manuscript.

COMPETING INTERESTS

SNR was a paid biomechanical consultant to SATech in 2008 for evaluating the force reduction provided by SmartCells flooring. He has no current links to the company. All other authors declare that they have no competing interests.

ETHICS APPROVAL

The protocol for this study was approved by the Research Ethics Boards at Simon Fraser University (2013s0525) and the Fraser Health Authority (FHREB 2012-059).

CONTRIBUTORS

CCL participated in study design, coordination and data collection, and helped to draft and revise the manuscript. FF helped to conceive the study, obtained funding, participated in study design and oversight, and helped to revise the manuscript. ACL helped to conceive of the study, participated in study design, and helped to revise the manuscript. PML participated in study design and oversight, coordination and data collection, and helped to revise the manuscript. SNR helped to conceive the study, obtained funding, participated in study design and oversight, and helped to revise the manuscript. DCM is the principal investigator and helped to conceive the study, obtained funding, participated in study design, coordination, data collection, statistical analysis planning, and oversight, and drafted and revised the manuscript. All authors read and approved the final manuscript.

REFERENCES

1. Grisso JA, Schwarz DF, Wishner AR, et al. Injuries in an elderly inner-city population. *J Am Geriatr Soc* 1990;38:1326-31.
2. Spaite DW, Criss EA, Valenzuela TD, et al. Geriatric injury: an analysis of prehospital demographics, mechanisms, and patterns. *Ann Emerg Med* 1990;19:1418-21.
3. Palvanen M, Kannus P, Parkkari J, et al. The injury mechanisms of osteoporotic upper extremity fractures among older adults: a controlled study of 287 consecutive patients and their 108 controls. *Osteoporos Int* 2000;11:822-31.
4. Harvey LA, Close JC. Traumatic brain injury in older adults: characteristics, causes and consequences. *Injury* 2012;43:1821-6.
5. Pickett W, Ardern C, Brison RJ. A population-based study of potential brain injuries requiring emergency care. *CMAJ* 2001;165:288-92
6. Empana JP, Dargent-Molina P, Breart G, et al. Effect of hip fracture on mortality in elderly women: the EPIDOS prospective study. *J Am Geriatr Soc* 2004;52:685-90.
7. Norton R, Butler M, Robinson E, et al. Declines in physical functioning attributable to hip fracture among older people: a follow-up study of case-control participants. *Disabil Rehabil* 2000;22:345-51.
8. Wolinsky FD, Fitzgerald JF, Stump TE. The effect of hip fracture on mortality, hospitalization, and functional status: a prospective study. *Am J Public Health* 1997;87:398-403.
9. Korhonen N, Niemi S, Parkkari J, et al. Incidence of fall-related traumatic brain injuries among older Finnish adults between 1970 and 2011. *JAMA* 2013;309:1891-2.

10. Watson WL, Mitchell R. Conflicting trends in fall-related injury hospitalisations among older people: variations by injury type. *Osteoporos Int* 2011;22:2623-31.

11. Thomas KE, Stevens JA, Sarmiento K, et al. Fall-related traumatic brain injury deaths and hospitalizations among older adults--United States, 2005. *J Safety Res* 2008;39:269-72.

12. Chesnut RM. Rehabilitation for traumatic brain injury: *Agency for Health Care Policy and Research* 1999.

13. Parachute. The cost of injury in Canada. Toronto, ON, 2015:26. http://parachutecanada.org/downloads/research/Cost_of_Injury-2015.pdf (accessed 24 Feb 2016).

14. Korall AM, Feldman F, Scott VJ, et al. Facilitators of and barriers to hip protector acceptance and adherence in long-term care facilities: a systematic review. *J Am Med Dir Assoc* 2015;16:185-93.

15. O'Loughlin JL, Robitaille Y, Boivin JF, et al. Incidence of and risk factors for falls and injurious falls among the community-dwelling elderly. *Am J Epidemiol* 1993;137:342-54.

16. Rubenstein LZ, Josephson KR, Robbins AS. Falls in the nursing home. *Ann Intern Med* 1994;121:442-51.

17. Tinetti ME, Speechley M, Ginter SF. Risk factors for falls among elderly persons living in the community. *N Engl J Med* 1988;319:1701-7.

18. Butler M, Norton R, Lee-Joe T, et al. The risks of hip fracture in older people from private homes and institutions. *Age Ageing* 1996;25:381-5.

19. Cameron ID, Gillespie LD, Robertson MC, et al. Interventions for preventing falls in older people in care facilities and hospitals. *Cochrane Database Syst Rev* 2012;12:CD005465

20. Vlaeyen E, Coussement J, Leysens G, et al. Characteristics and effectiveness of fall prevention programs in nursing homes: a systematic review and meta-analysis of randomized controlled trials. *J Am Geriatr Soc* 2015;63:211-21.

21. Vu MQ, Weintraub N, Rubenstein LZ. Falls in the nursing home: are they preventable? *J Am Med Dir Assoc* 2006;7:S53-8.

22. Papaioannou A, Santesso N, Morin SN, et al. Recommendations for preventing fracture in long-term care. *CMAJ* 2015;187:1135-44.

23. Santesso N, Carrasco-Labra A, Brignardello-Petersen R. Hip protectors for preventing hip fractures in older people. *Cochrane Database Syst Rev* 2014;3:CD001255.

24. Laing AC, Robinovitch SN. Low stiffness floors can attenuate fall-related femoral impact forces by up to 50% without substantially impairing balance in older women. *Accid Anal Prev* 2009;41:642-50.

25. Laing AC, Tootoonchi I, Hulme PA, et al. Effect of compliant flooring on impact force during falls on the hip. *J Orthop Res* 2006;24:1405-11.

26. Maki BE, Fernie GR. Impact attenuation of floor coverings in simulated falling accidents. *Appl Ergon* 1990;21:107-14.

27. Simpson AH, Lamb S, Roberts PJ, et al. Does the type of flooring affect the risk of hip fracture? *Age Ageing* 2004;33:242-6.

28. Wright AD, Laing AC. The influence of headform orientation and flooring systems on impact dynamics during simulated fall-related head impacts. *Med Eng Phys* 2012;34:1071-8.

29. Glinka MN, Karakolis T, Callaghan JP, et al. Characterization of the protective capacity of flooring systems using force-deflection profiling. *Med Eng Phys* 2013;35:108-15.

30. Glinka MN, Cheema KP, Robinovitch SN, et al. Quantification of the trade-off between force attenuation and balance impairment in the design of compliant safety floors. *J Appl Biomech* 2013;29:563-72.

31. Wright AD, Heckman GA, McIlroy WE, et al. Novel safety floors do not influence early compensatory balance reactions in older adults. *Gait Posture* 2014;40:160-5.

32. Wright AD, Laing AC. The influence of novel compliant floors on balance control in elderly women--A biomechanical study. *Accid Anal Prev* 2011;43:1480-7.

33. Bhan S, Levine I, Laing AC. The influence of body mass index and gender on the impact attenuation properties of flooring systems. *J Appl Biomech* 2013;29:731-9.

34. Bhan S, Levine IC, Laing AC. Energy absorption during impact on the proximal femur is affected by body mass index and flooring surface*. J Biomech* 2014;47:2391-7.

35. Choi WJ, Hoffer JA, Robinovitch SN. Effect of hip protectors, falling angle and body mass index on pressure distribution over the hip during simulated falls. *Clin Biomech* 2010;25:63-9.

36. Knoefel F, Patrick L, Taylor J, et al. Dual-stiffness flooring: can it reduce fracture rates associated with falls? *J Am Med Dir Assoc* 2013;14:303-5.

37. Gustavsson J, Bonander C, Andersson R, et al. Investigating the fall-injury reducing effect of impact absorbing flooring among female nursing home residents: initial results. *Inj Prev* 2015;21:320-4.

38. Drahota AK, Ward D, Udell JE, et al. Pilot cluster randomised controlled trial of flooring to reduce injuries from falls in wards for older people. *Age Ageing* 2013;42:633-40.

39. Hanger HC, Hurley K, White A. Low impact flooring - does it reduce falls or injuries [*abstract*]? 6th Australian and New Zealand Falls Prevention Conference 2014.

40. Rapp K, Becker C, Cameron ID, et al. Epidemiology of falls in residential aged care: analysis of more than 70,000 falls from residents of bavarian nursing homes. *J Am Med Dir Assoc* 2012;13:187 e1-6.

41. Lamb SE, Jorstad-Stein EC, Hauer K, et al. Development of a common outcome data set for fall injury prevention trials: the Prevention of Falls Network Europe consensus. *J Am Geriatr Soc* 2005;53:1618-22.

42. Hawes C, Morris JN, Phillips CD, et al. Development of the nursing home Resident Assessment Instrument in the USA. *Age Ageing* 1997;26 Suppl 2:19-25.

43. Morris JN, Hawes C, Fries BE, et al. Designing the national resident assessment instrument for nursing homes. *Gerontologist* 1990;30:293-307.

44. Hanger HC, Hurley K, Hurring S, et al. Low impact flooring - is it practical in a hospital [*abstract*]? 6th Australian and New Zealand Falls Prevention Conference 2014.

45. Schwenk M, Lauenroth A, Stock C, et al. Definitions and methods of measuring and reporting on injurious falls in randomised controlled fall prevention trials: a systematic review. *BMC Med Res Methodol* 2012;12:50.

46. Ray WA, Taylor JA, Brown AK, et al. Prevention of fall-related injuries in long-term care: a randomized controlled trial of staff education*. Arch Intern Med* 2005;165:2293-8.

47. Campbell AJ, Robertson MC, Gardner MM, et al. Randomised controlled trial of a general practice programme of home based exercise to prevent falls in elderly women. *BMJ* 1997;315:1065-9.

48. Schonnop R, Yang Y, Feldman F, et al. Prevalence of and factors associated with head impact during falls in older adults in long-term care. *CMAJ* 2013;185:E803-10.

49. Karon SL, Lazarus JA, Holman L. Challenges and approaches to the identification of traumatic brain injury among nursing home residents. *J Head Trauma Rehabil* 2007;22:350-9.

FIGURE LEGENDS

Figure 1. Flow chart describing the design of the Flooring for Injury Prevention (FLIP) Study.

Figure 2. FLIP Study protocol for ascertaining primary (serious fall-related injury) and secondary (minor fall-related injury) outcomes.

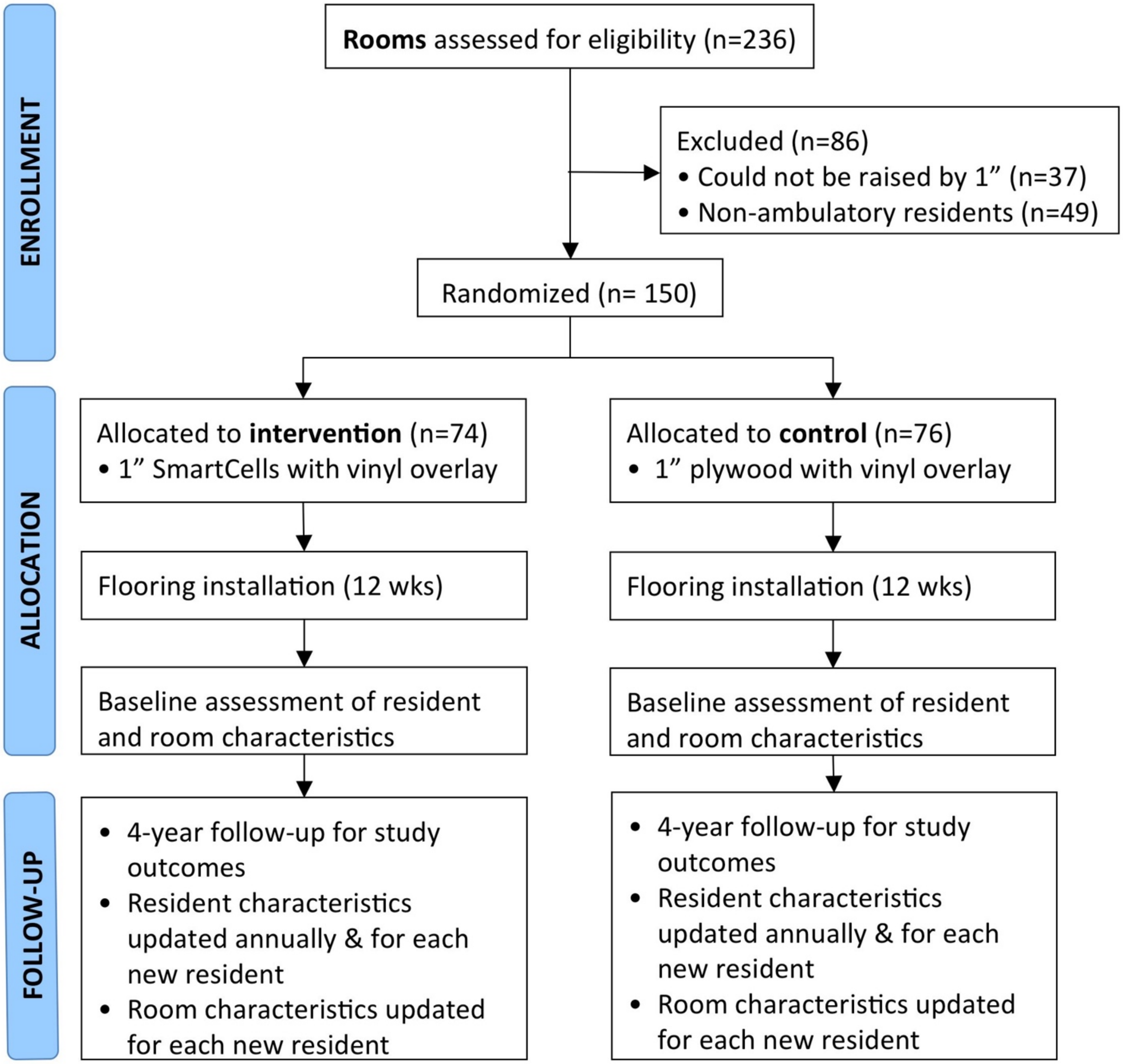

ENROLLMENT
Rooms assessed for eligibility (n=236)
Excluded (n=86)
• Could not be raised by 1” (n=37)
• Non-ambulatory residents (n=49)
Randomized (n= 150)
ALLOCATION
Allocated to intervention (n=74)
• 1” SmartCells with vinyl overlay
Allocated to control (n=76)
• 1” plywood with vinyl overlay
Flooring installation (12 wks)
Flooring installation (12 wks)
Baseline assessment of resident and room characteristics
Baseline assessment of resident and room characteristics
FOLLOW-UP
• 4-year follow-up for study outcomes
• Resident characteristics updated annually & for each new resident
• Room characteristics updated for each new resident
• 4-year follow-up for study outcomes
• Resident characteristics updated annually & for each new resident
• Room characteristics updated for each new resident

Figure 1.

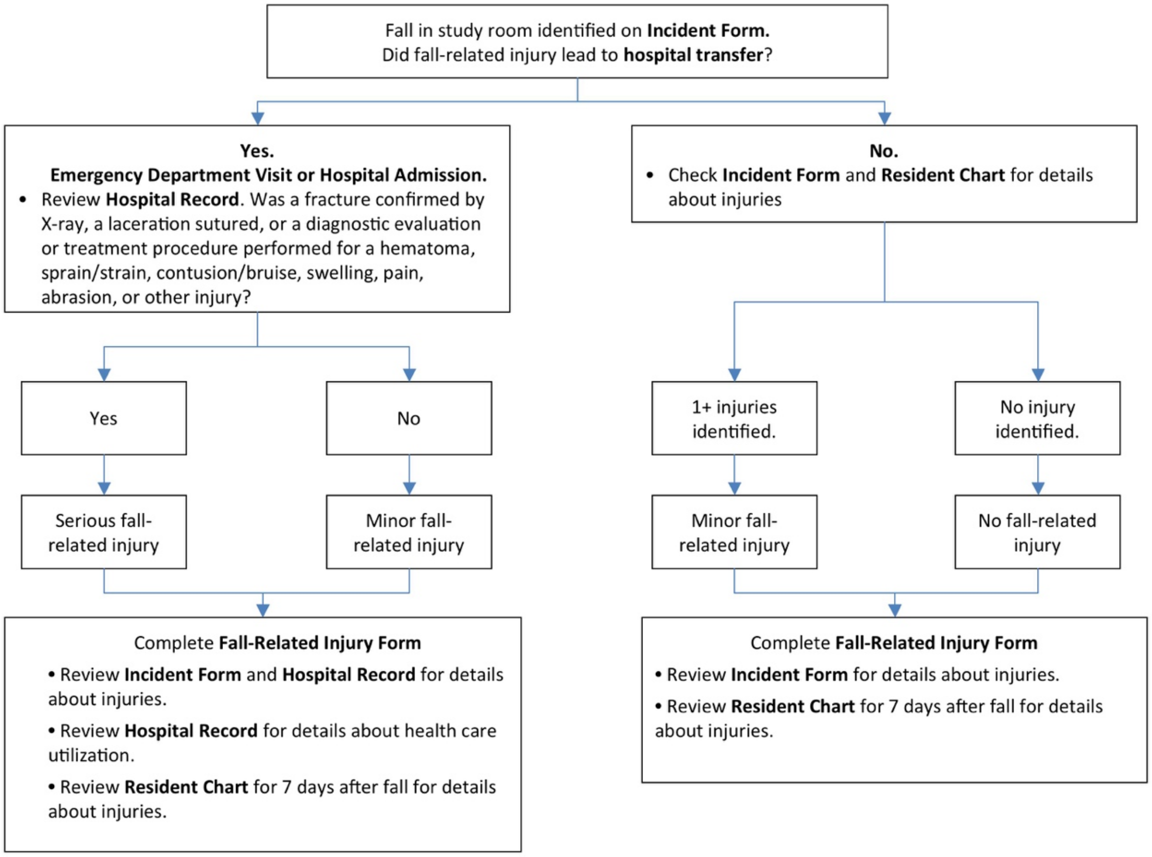

Fall in study room identified on Incident Form.
Did fall-related injury lead to hospital transfer?
Yes.
Emergency Department Visit or Hospital Admission.
• Review Hospital Record. Was a fracture confirmed by X-ray, a laceration sutured, or a diagnostic evaluation or treatment procedure performed for a hematoma, sprain/strain, contusion/bruise, swelling, pain, abrasion, or other injury?
No.
• Check Incident Form and Resident Chart for details about injuries
Yes
No
1+ injuries identified.
No injury identified.
Serious fall-related injury
Minor fall-related injury
Minor fall-related injury
No fall-related injury
Complete Fall-Related Injury Form
• Review Incident Form and Hospital Record for details about injuries.
• Review Hospital Record for details about health care utilization.
• Review Resident Chart for 7 days after fall for details about injuries.
Complete Fall-Related Injury Form
• Review Incident Form for details about injuries.
• Review Resident Chart for 7 days after fall for details about injuries.

Figure 2.
